# Deep‐targeted gene sequencing reveals ARID1A mutation as an important driver of glioblastoma

**DOI:** 10.1111/cns.14698

**Published:** 2024-04-11

**Authors:** Menglin Xiao, Xiaoteng Cui, Can Xu, Lei Xin, Jixing Zhao, Shixue Yang, Biao Hong, Yanli Tan, Jie Zhang, Xiang Li, Jie Li, Chunsheng Kang, Chuan Fang

**Affiliations:** ^1^ Department of Neurosurgery Affiliated Hospital of Hebei University Baoding China; ^2^ Hebei Key Laboratory of Precise Diagnosis and Treatment of Glioma Baoding China; ^3^ Laboratory of Neuro‐oncology Tianjin Neurological Institute, Tianjin Medical University General Hospital Tianjin China; ^4^ Department of Pathology Affiliated Hospital of Hebei University Baoding China; ^5^ Department of Pathology Hebei University School of Basic Medical Sciences Baoding China; ^6^ Department of Proteomics Tianjin Enterprise Key Laboratory of Clinical Multi‐omics Tianjin China

**Keywords:** ARID1A, chemoresistance, deep targeted sequencing, DNA damage repair, glioblastoma

## Abstract

**Aims:**

To investigate the key factors influencing glioma progression and the emergence of treatment resistance by examining the intrinsic connection between mutations in DNA damage and repair‐related genes and the development of chemoresistance in gliomas.

**Methods:**

We conducted a comprehensive analysis of deep‐targeted gene sequencing data from 228 glioma samples. This involved identifying differentially mutated genes across various glioma grades, assessing their functions, and employing I‐TASSER for homology modeling. We elucidated the functional changes induced by high‐frequency site mutations in these genes and investigated their impact on glioma progression.

**Results:**

The analysis of sequencing mutation results of deep targeted genes in integration revealed that ARID1A gene mutation occurs frequently in glioblastoma and alteration of ARID1A could affect the tolerance of glioma cells to temozolomide treatment. The deletion of proline at position 16 in the ARID1A protein affected the stability of binding of the SWI/SNF core subunit BRG1, which in turn affected the stability of the SWI/SNF complex and led to altered histone modifications in the CDKN1A promoter region, thereby affecting the biological activity of glioma cells, as inferred from modeling and protein interaction analysis.

**Conclusion:**

The ARID1A gene is a critical predictive biomarker for glioma. Mutations at the ARID1A locus alter the stability of the SWI/SNF complex, leading to changes in transcriptional regulation in glioma cells. This contributes to an increased malignant phenotype of GBM and plays a pivotal role in mediating chemoresistance.

## Graphical abstract

Deep sequencing targeting DNA damage repair pathways identifies ARID1A as a prognostic biomarker for gliomas and its mutation alters SWI/SNF stability increasing the malignant phenotype of GBM and mediating chemoresistance.

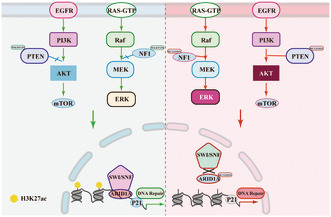

## 1 INTRODUCTION

Gliomas are the most common primary malignant brain tumor, accounting for 80% of primary central nervous system malignancies. Despite continuous improvements in current treatment modalities, progression‐free survival (PFS) for WHO grade 4 glioblastoma multiforme (GBM) remains relatively short.^1^ This short survival may be related to the rapid transformation of GBM into a chemotherapy‐resistant malignancy after temozolomide (TMZ) treatment, which in turn leads to the phenomenon of recurrent genetic heterogeneity.^2^ It has been shown that genome‐wide DNA alkylation mediated by temozolomide, an “orphan” chemotherapeutic agent currently used in the treatment of glioma, activates the DNA mismatch repair (MMR) pathway.^3^ This pathway involves the formation of lethal DNA double‐strand breaks (DSBs) at mismatch sites and two repair mechanisms, non‐homologous recombination joining (NHEJ) and homologous recombination repair (HR).^4^ The unstable nature of the genome due to DNA mismatch repair is often considered to be a key driver of tumor genesis, cancer recurrence, and chemotherapy‐resistant metastasis.^5^ However, the intrinsic link between altered expression profiles caused by mutations in DNA repair‐associated genes and GBM chemoresistance in gliomas remains understudied and poorly understood. Therefore, mechanistic studies on this issue deserve further in‐depth investigation to improve our understanding of therapeutic resistance in gliomas.

With the progressive implementation of the Human Genome Project, the new World Health Organization (WHO) classification of central nervous system (CNS) tumors has shifted from a histology‐based approach to a combination of histological and molecular features to reduce discrepancies in pathological examination.^6^ Integrated DNA sequencing and copy number analysis have identified potential driver genes, and molecular profiling, as demonstrated by several clinical sequencing efforts, not only provides insights into glioma biology, but also offers multi‐level solutions, including the development of novel small molecule drugs.^7^, ^8^ Targeted sequencing is the selection of mostly established cancer genes for targeted sequencing of the disease to facilitate the selection of the most likely responders to certain anticancer drugs.^9^ Deep targeted sequencing of tumor samples is becoming increasingly valuable and its application in the clinic deserves further investigation.

In this study, we focused on investigating DNA damage and repair (DDR) and altered expression of genes associated with genomic instability. A total of 228 pairs of glioma samples from two cohorts were analyzed by deep‐targeted gene sequencing, which revealed a different spectrum of mutations between high‐grade and low‐grade gliomas, as well as changes in protein structure caused by the mutations. Based on this result, a series of in vitro and in vivo experiments were performed and it was found that mutations in the ARID1A gene may be a key molecular event in GBM. In addition, the mutation in the 16th amino acid of the ARID1A protein reduced the activity of the SWI/SNF complex by affecting the stability of BRG1, which may promote glioma progression and the development of TMZ resistance.

## 2 RESULTS

### 2.1 Targeted gene sequencing analysis of different grade glioma cohorts reveals a high frequency of ARID1A mutations in GBM

**FIGURE 1:**
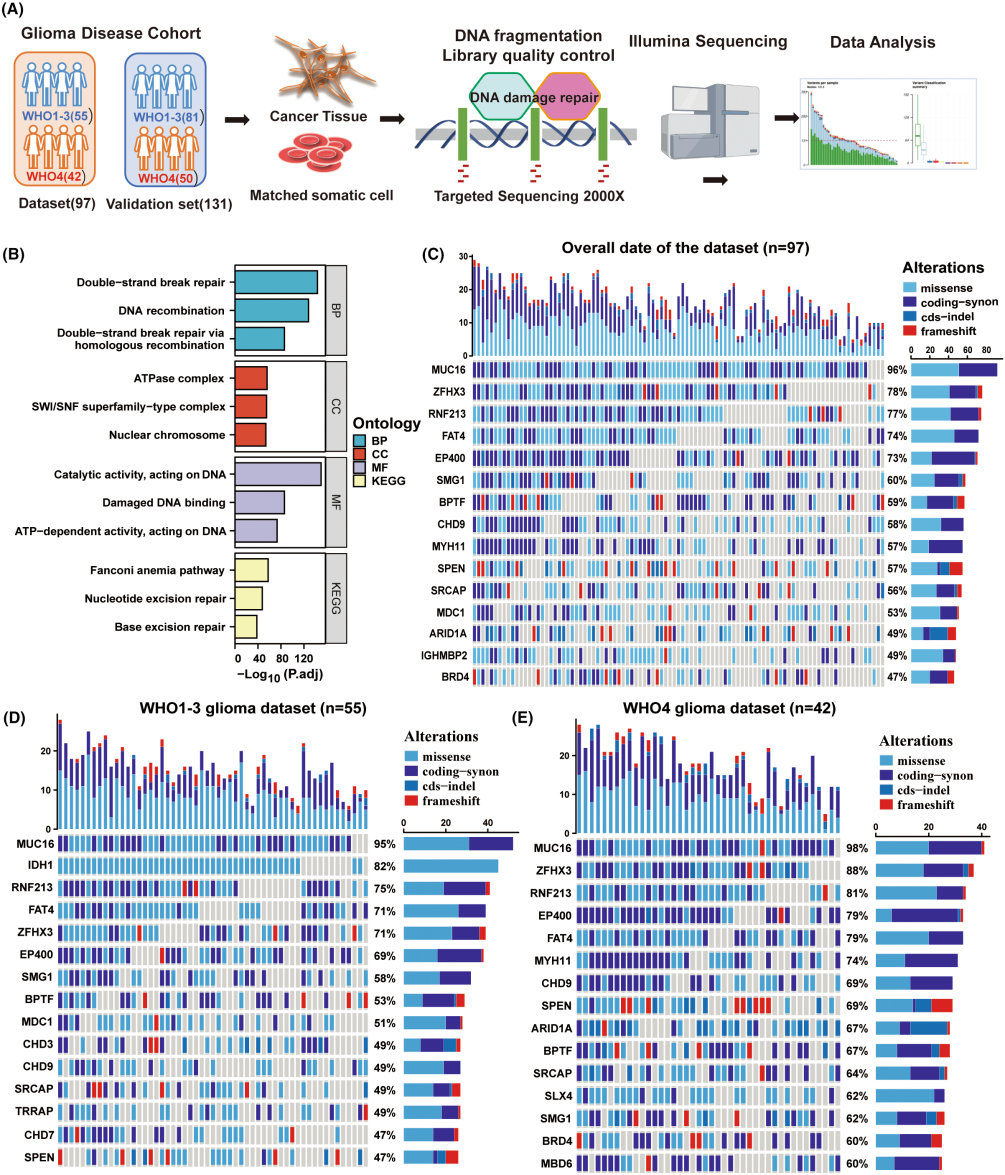
Genomic analysis of the glioma cohort identifies frequent ARID1A mutations in GBM. (A) Targeted gene proteomics workflow overview. (B) GO and KEGG analysis of 428 genes by deep sequencing, focusing on the cell cycle, chromatin stabilization, and DNA damage and repair (DDR) pathways. (C) Waterfall plot of the 15 genes with the highest mutation frequency in the glioma dataset (*n* = 97). (D) Waterfall plot of the 15 genes with the highest mutation frequency in the WHO grades 1–3 glioma dataset (*n* = 55). (E) Waterfall plot of the 15 genes with the highest mutation frequency in the WHO grade 4 glioma dataset (*n* = 42).

To investigate the variability of DNA damage and repair (DDR)‐related genes in glioma patients and to identify potential biomarkers, we sequenced and analyzed the related genes using the PE150 sequencing method in combination with a depth of 2000× targeted sequencing technology. The workflow diagram of this study is shown in Figure 1A. Our main objective was to perform data analysis on the dataset and validation cohort (Table 1) to identify possible mutational signatures. For this purpose, we carefully selected 428 genes associated with DDR and genomic instability pathways for deep sequencing analysis. The protein products encoded by these genes form a network centered on key cellular processes such as cell cycle regulation, chromatin stability and DDR (Figure 1B). We further screened the sequencing results for mutations that could alter the conformation of the proteins encoded by the genes, including missense mutations, coding mutations, cds‐indel mutations and code‐shifting mutations. We identified the top 15 disease‐causing mutations with the highest mutation rates in the entire glioma patient cohort, including MUC16, ZFHX3, RNF214, FAT4, EP400, SMG1, BPTF, CHD9, MYH11, SPEN, SRCAP, MDC1, ARID1A, and BRD4 (Figure 1C). In parallel, we correlated the results of our sequencing analyses with those of the TCGA database and the GBM dataset in the CGGA database (Figure S1A,B).

**TABLE 1:** Basic characteristics of cohort patients.

Dataset cohort (*n* = 97)		
Variable	WHO4 (*n* = 42)	WHO1‐3 (*n* = 55)
Age‐mean, year (range)	55.7 (30–74)	45.25 (11–67)
Male	31 (73.8%)	26 (46.4%)
IDH1 wildtype	42 (100%)	10 (18.2%)
Primary	34 (80.9%)	48 (87.3%)
Recurrent	8 (19.1%)	7 (12.7%)

Validation cohort (*n* = 131)		
Variable	WHO4 (*n* = 49)	WHO1‐3 (*n* = 82)
Age‐mean, year (range)	51.3 (36–72)	48.5 (16–65)
Male	28 (57.1%)	45 (54.9%)
IDH1 wildtype	49 (100%)	8 (9.8%)
Primary	42 (85.7%)	74 (90.2%)
Recurrent	7 (14.3%)	8 (9.8%)

The results of the deep sequencing analyses showed impressive features, with the increased sequencing depth revealing potential molecular variants not detected by conventional sequencing methods, such as ZFHX3, RNF214, SPEN, SRCAP, MDC1, ARID1A, BRD4, and so on. We then applied the World Health Organization (WHO) classification of central nervous system (CNS) tumors to classify the tumor samples into two grades, that is, WHO grade 4 and WHO grades 1–3, and grouped the data with the clinicopathological diagnostic reports of the two cohorts. By comparing the results of the first 15 mutation percentages, we found that the mutation frequency of ARID1A in WHO grade 1–3 (Figure 1D) (36%, not in the first 15) was significantly lower than that of ARID1A in WHO grade 4 (Figure 1E) (67%). The mutation frequency of high‐frequency mutated genes in the WHO grade 4 glioma patients in the validation cohort was broadly similar to the results in the dataset, with a mutation frequency of 63% in ARID1A (Figure S1C). In conclusion, these results suggest that ARID1A mutations may be more common in WHO grade 4 gliomas and that deep‐targeted gene sequencing may provide valuable clues for further investigation of glioblastoma biomarkers in the future.

### 2.2 ARID1A mutation is an essential molecular event in GBM pathogenesis

Since the conventional whole‐exome sequencing results provided less information about mutations in the ARID1A gene and that gene‐damaging mutations usually involve dysregulation of the expression of gene–protein functions. Therefore, we used Matascape to perform GO and KEGG pathway analysis of the genes that were negatively correlated (*R* < −0.6) with ARID1A expression in the GBM gene expression profile in the TCGA database. Targeted deep sequencing revealed that the ARID1A gene was co‐occurring with the TP53 and BPTF genes, but not mutually exclusive with the other genes (Figure S1D), suggesting that the mutation in ARID1A was independent of the other genes. Moreover, univariate Cox regression analysis from the cohorts suggested that the mutation of ARID1A, older age, chemotherapy, and radiotherapy were associated with overall survival outcomes. Further analysis using the multivariate Cox regression analysis revealed that the mutation of ARID1A was correlated with overall survival (Figure S1E). The results of the gene enrichment analysis showed that these genes were mainly enriched in signaling pathways such as oxidative phosphorylation, mitochondrial complex IV assembly, and proton transmembrane transport (Figure S1F).

**FIGURE 2:**
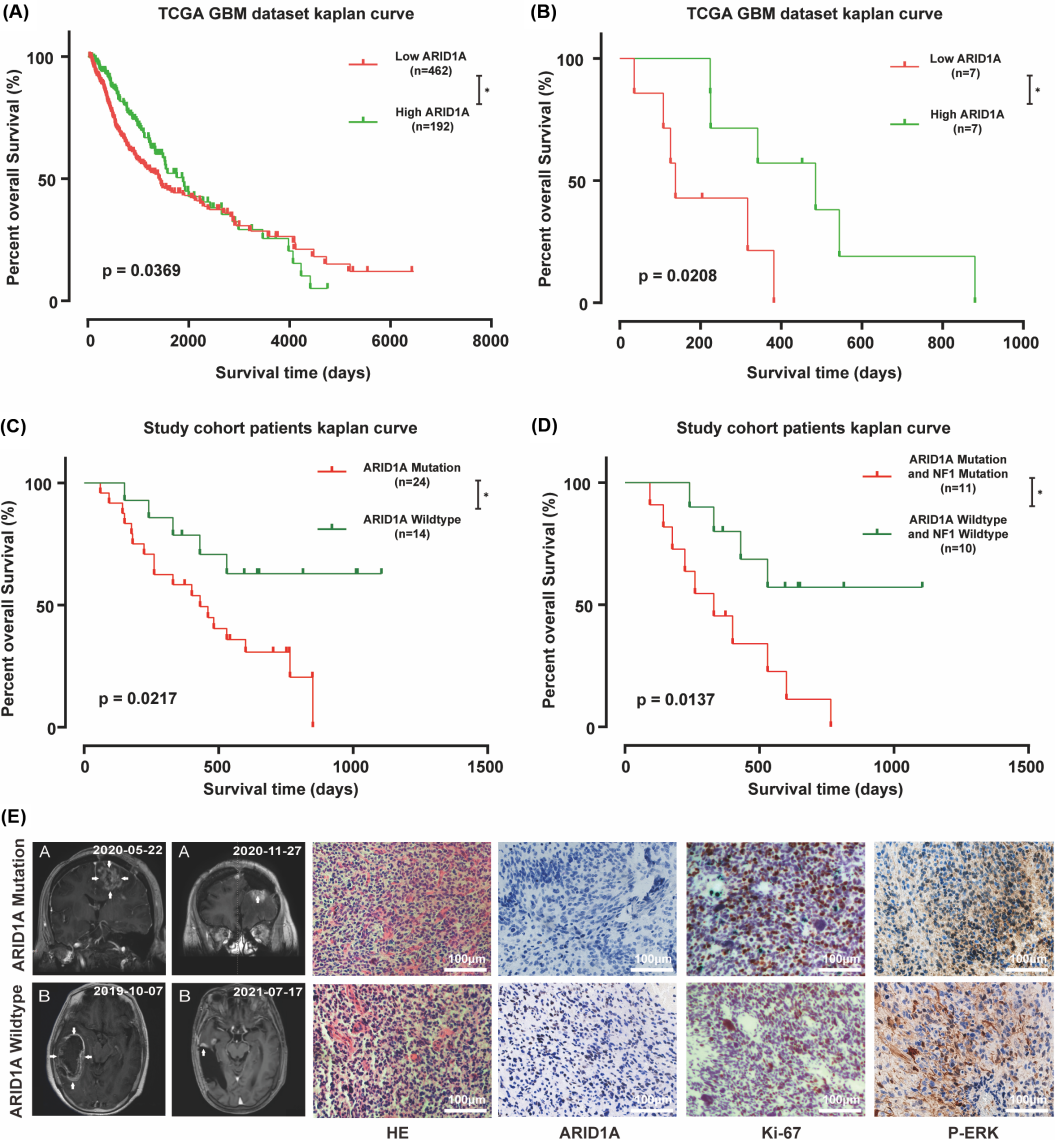
ARID1A mutation is an essential molecular event in GBM pathogenesis. (A) Kaplan–Meier curve analysis showed that patients with low ARID1A scores in the TCGA cohort had a poor prognosis. *p = 0.0369 (Log‐rank test). (B) Kaplan–Meier curve analysis indicated that patients with low ARID1A scores in the TCGA GBM cohort with NF1 mutation had a poor prognosis. *p = 0.0208 (Log‐rank test). (C) Kaplan–Meier curve analysis showed that patients with ARID1A mutations in the dataset cohort had a poor prognosis. *p = 0.0217 (Log‐rank test). (D) Kaplan–Meier curve analysis showed that patients with co‐mutations in NF1 and ARID1A in the dataset cohort had a worse prognosis than patients with either NF1 or ARID1A mutation. *p = 0.0137 (Log‐rank test). (E) A comparison of imaging and postoperative H&E and immunohistochemical (IHC) findings in two representative patients at the time of initial surgery and after recurrence or at the last follow‐up visit. Patients with ARID1A mutation presented a higher malignant phenotype. Scale bar = 100 μm.

We further analyzed the relationship between patient survival and ARID1A gene expression in the TCGA GBM database and found that the prognosis of GBM patients was positively correlated with the expression level of ARID1A (*p* = 0.0369) (Figure 2A). In addition, in the TCGA GBM patient database, the mutation status of the NF1 gene also affected the relationship between ARID1A expression and patient prognosis: the lower the ARID1A expression level in patients with NF1 mutations, the worse the survival of the patients (*p* = 0.0208) (Figure 2B). In our sequencing study cohort, we analyzed the relationship between ARID1A mutations and the prognosis of glioma patients and found that ARID1A mutations were negatively correlated with the prognosis of glioma patients (*p* = 0.0217) (Figure 2C). Further joint analysis of the mutation status of NF1 revealed that the survival prediction of patients with co‐mutation of NF1 and ARID1A was worse than that of patients with both genes wild‐type (*p* = 0.0137) (Figure 2D). These results suggest that the expression level and mutation status of ARID1A can provide further prognostic analysis for conventional glioma prognostic information to guide patient treatment.

In conjunction with the results of deep targeted gene sequencing, we selected two representative patients from our clinical sample, one with wild‐type ARID1A and the other with mutant ARID1A. Both patients underwent total tumor resection, and the resection specimens were analyzed in four independent directions with the same mutational load in the four tumor regions, which was validated by using the sequencing data from the clinical patients and comparing the results of nuclear magnetic imaging after the initial surgery and at the time of recurrence or the last follow‐up, based on the histopathological and immunohistochemical (IHC) analysis of the expression of the ARID1A gene in relation to the tumor samples, and the ability of the tumors to proliferate and the correlation between the Ras/Raf/MEK/ERK pathway was analyzed, and we observed higher Ki‐67 and p‐ERK expression in patients with ARID1A mutations (Figure 2E), the results of the statistical analysis are presented in Figure S2A, suggesting that the mutation may be associated with hyperactivation of the RAF pathway. We used GSEA analysis to compare the Ras/Raf/MEK/ERK gene expression profiles of wild‐type samples and ARID1A mutant samples from the CGGA database to identify potential pathways regulated by ARID1A status RAF. The expression pattern of the set of genes involved in apoptosis (Figure S2B) and TNFA signaling via NFKB (Figure S2C) was closer to the phenotype of the wild‐type sample set, and to validate this finding we increased the number of clinical samples for immunohistochemical experiments, and there was higher expression of p‐MEK1\2 in the ARID1A mutation glioblastoma samples (Figure S2D,E). Taken together, these results suggest that mutations in the ARID1A gene may be an important molecular event in GBM, which may be associated with aberrant activation of the Ras/Raf/MEK/ERK pathway.

### 2.3 Downregulated expression of ARID1A promotes malignant behavior of GBM cells and TMZ resistance

**FIGURE 3:**
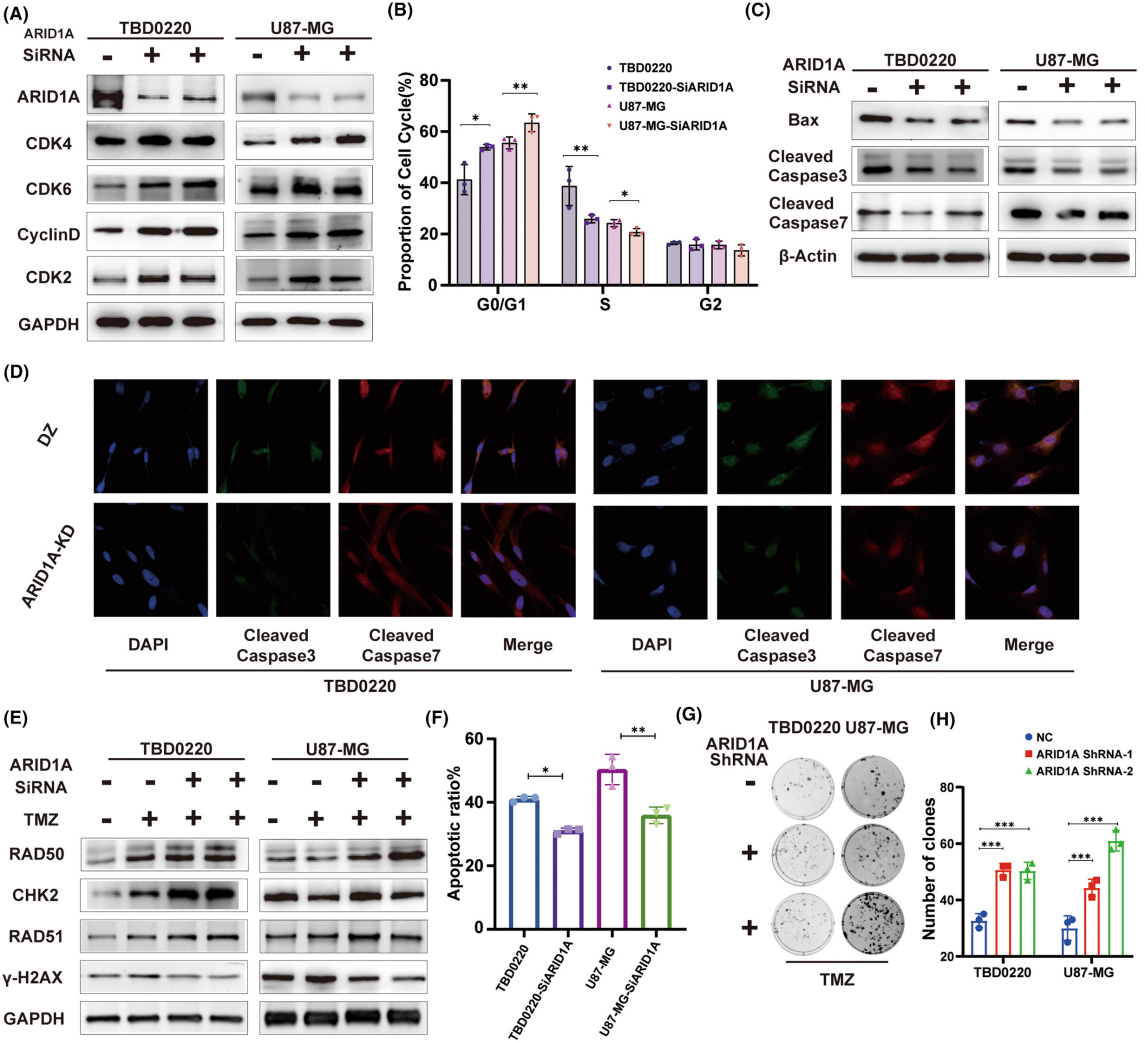
The function of ARID1A in glioma cells in vitro. (A) TBD0220 and U87‐MG cell lines were used to interfere with the expression of ARID1A by ShRNA treatment. The expression levels of cell cycle‐related proteins, including CDK4, CDK6, CyclinD, and CDK2, were detected by western blotting (WB). GAPDH served as the loading control. (B) Flow cytometric analysis of G1‐phase arrest in TBD0220 and U87‐MG cells. (C) WB analysis of BAX, Caspase‐3/7and β‐actin expressions in TBD0220, and U87‐MG cell lines. (D) Immunofluorescence (IF) assay for Caspase‐3 and Caspase‐7 expressions. (E) WB analysis to measure levels of RAD50, CHK2, RAD51, and γH2AX in TBD0220, and U87‐MG cell lines treated with DMSO or 200 μM of TMZ. (F) Flow cytometric analysis of apoptosis in differentially expressing ARID1A in TBD0220, and U87‐MG cell lines, following exposure to 200 μM of TMZ. (G) Colony formation assays in GBM cell lines treated with 150 μM of TMZ or DMSO. (H) A colony formation assay was performed in GBM cells. Data are represented as the mean ± SEM (*n* = 3). *p < 0.05, **p < 0.01, ***p < 0.001, ns represents *p* > 0.05.

In order to deeply investigate the function of ARID1A in glioma (GBM) cells, we first explored the relationship between ARID1A and the malignant biological behaviors of GBM by characterizing cell cycle‐related proteins. We selected the U87‐MG and TBD0220 cell lines for validation. RNA interference‐mediated knockdown (Knockdown, KD) of ARID1A in GBM cell lines was verified by qRT‐PCR (Figure S3A) and immunoblotting (WB). Previous immunohistochemistry (IHC) results of clinical samples showed that the expression of ARID1A was negatively correlated with the expression of the cell proliferation marker Ki‐67. The results showed that the expression levels of CDK4, CDK6, Cyclin D1, and CDK2 were higher in the ARID1A KD cell line compared to the control group (Figure 3A). Downregulation of ARID1A significantly promoted the expression of glioma cell cycle‐related proteins and accelerated cell cycle progression. Flow cytometry analysis showed that the G0/G1 phase ratio of GBM cells increased after ARID1A KD (Figure 3B), further elucidating the association between ARID1A and cell cycle. In addition, WB results showed that ARID1A KD significantly decreased the levels of BAX (a key component of apoptosis in GBM cells) and caspase‐3/7 (downstream effector proteins) (Figure 3C). Subsequently, confocal microscopy further confirmed this (Figure 3D). These results suggest that reduced expression of ARID1A may lead to reduced apoptosis and accelerated cell cycle, thereby promoting the malignant phenotype of glioma cells.

We conducted a further analysis of the impact of ARID1A mutation on DDR pathway factors following treatment with 200 μM TMZ, in comparison to wild‐type ARID1A cells. The levels of DDR proteins including RAD50, RAD51, and CHK2 were upregulated and γH2AX levels were downregulated after TMZ exposure in GBM cells (Figure 3E). Flow cytometric analysis showed that apoptosis was significantly reduced in GBM cells after ARID1A KD in response to TMZ treatment (Figure 3F). Furthermore, ARID1A KD cells demonstrated significant clonal growth following the treatment regimen of 150 μM TMZ, as compared to control cells (Figure 3G,H). In conclusion, ARID1A may regulate the cell cycle state and apoptosis of GBM cells, and ARID1A KD may enhance the therapeutic resistance response of GBM to TMZ, thereby promoting the ability of glioma cells to resist TMZ in vitro.

### 2.4 Mutations in amino acid 16 of the ARID1A protein can affect the stability of the SWI/SNF complex

**FIGURE 4:**
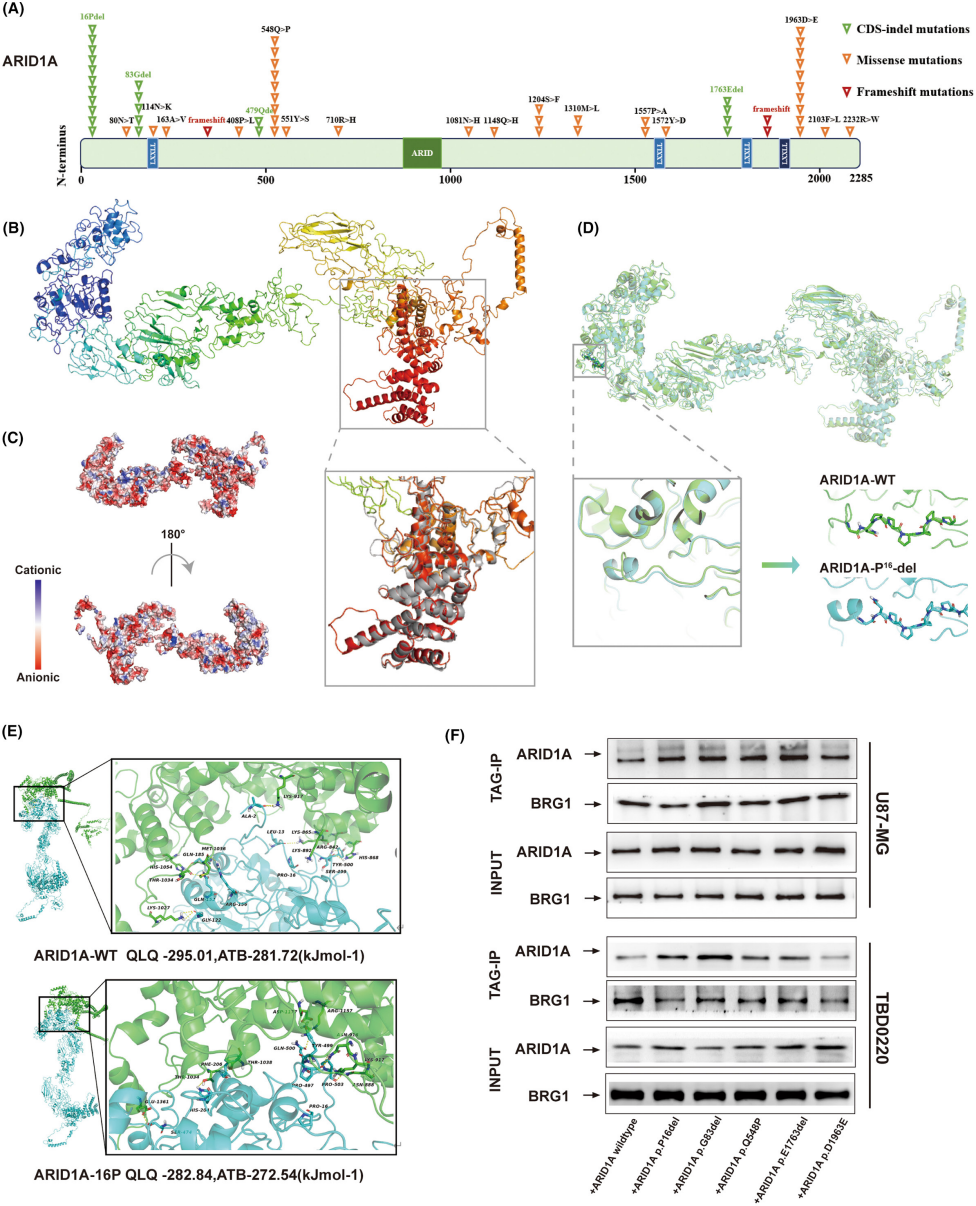
Mutation of the 16th amino acid of the ARID1A protein affects the stability of the SWI/SNF complex. (A) Schematic representation of protein loci of ARID1A in GBM by deep sequencing. (B) Multi‐template homology modeling uncovered the three‐dimensional (3D) structural simulation of the ARID1A protein. (C) The surface of ARID1A was widely negatively charged, indicating a possible structural foundation for protein–protein interactions. (D) Mutations in the ARID1A gene affected its local 3D conformations. (E) The 3D structure of the ATP hydrolase structural domain of the BRG1 protein bound to ARID1A‐WT and ARID1A‐P16 variants. (F) Co‐IP experiments were performed using TBD0220 and U87‐MG cell extracts revealing that the P16 deletion site could bind a relatively smaller number of BRG1 protein molecules compared to other mutants and wild‐type forms.

Through in‐depth deep targeted gene sequencing analysis of glioma (GBM) tissues, we identified five high‐frequency loci for mutations in the ARID1A gene in GBM patients (Figure 4A). Disruptive mutations can lead to amino acid alterations, which in turn affect changes in protein structure. Therefore, we first determined the three‐dimensional structure of the ARID1A protein, which contains an AT‐rich interacting structural domain (ARID), which is essential for DNA binding of the ARID1A protein, a nuclear localization signal (NLS) sequence and three LxxLL motifs, which are capable of interacting with hormone receptors. Hydrophilicity analysis showed that the content of hydrophilic residues in ARID1A was about 93.3% of the total amino acid residues, with a hydrophilic value of −0.778 (Figure S4A). Then, we predicted the multi‐template homology using I‐TASSER and RoseTTAFold methods, and selected the model with the highest score for secondary modeling to obtain the complete protein structure. Next, the optimized ARID1A structure was obtained using GROMACS 2018 kinetics software (Figure 4B). The results showed that the structure of ARID1A consisted of helices, folded chains and random loops, in which its conserved structural domains were highly overlapped with the template (as shown in the gray box of Figure 4B). ARID1A had a negative charge on its surface, which provided a possible structural basis for protein–protein interactions (Figure 4C). Thus, we resolved the three‐dimensional structure of the full‐length ARID1A protein, providing a structural basis for its interaction with partner proteins.

Next, we sought to analyze whether the five high‐frequency site mutations identified in deep‐targeted gene sequencing have an effect on the ARID1A structure in comparison with the wild‐type ARID1A structure. Based on the structural model of wild‐type (WT) ARID1A, we simulated the structure of mutant ARID1A with P16, G83, and E1763 deletions as well as the Q548P and D1963E mutations, respectively. We superimposed each optimized mutant structure with the WT model of the ARID1A protein to analyze the conformational changes. We further performed an unfolded amplification study of the mutant regions with highly overlapping structures. We found that the deletion of P16 reduced the length of the loop structure (Figure 4D) and converted it into a small helical chain, leading to altered stability of the chain structure, which may affect the biological activity of the ARID1A protein. The results of structural changes due to mutations at other sites are shown in Figure S4B–E.

To further investigate whether site mutations affect the stability of the SWI/SNF complex, we obtained the amino acid sequence of the main component of the complex BRG1 and predicted its 3D structure using templates from the Protein Data Bank (PDB). Based on the NCBI blast search results (Table S1), we identified template structures with high similarity to BRG1, including 7VDV, 6UXV, 5HZR, 7EGM, and 5X0X. We chose 7VDV with the highest template score to perform single‐template homology modeling and optimized the three‐dimensional structure of BRG1 using the GROMACS 2018 dynamics software (Figure S4F). The constructed three‐dimensional structures of the target proteins were found to be accurate as verified by Ramachandran analysis and MODELER construction.

By ab initio free docking analysis, we compared the interaction between the receptor protein BRG1 and wild‐type (WT) ARID1A as well as P16 mutant ARID1A. The results showed that WT ARID1A had higher stability and biological activity upon binding to BRG1 compared to the P16 mutant. Indeed, the ARID1A‐P16 deletion effectively blocked the binding of ARID1A to BRG1, attenuated the overall stability of the SWI/SNF complex, and altered the biological activity of the complex (Figure 4E).

To further confirm our findings, we designed the ARID1A gene mutation site plasmid identified in deep targeted gene sequencing and performed qRT‐PCR to verify the transfection efficiency (Figure S3B). Next, we performed Co‐IP experiments using U87‐MG and TBD0220 cell extracts (Figure 4F), and the results confirmed that the binding rate of the P16 deletion site to the BRG1 protein was lower than that of the other sites and the WT variant, which was consistent with our previous structural modeling results. Thus, our results suggest that the ARID1A‐P16 deletion mutation affects its binding to BRG1 and attenuates the overall stability of the SWI/SNF complex.

### 2.5 P16 deletion of ARID1A affects the stability of the SWI/SNF complex, further promoting the TMZ resistance in glioma cells in vitro

**FIGURE 5:**
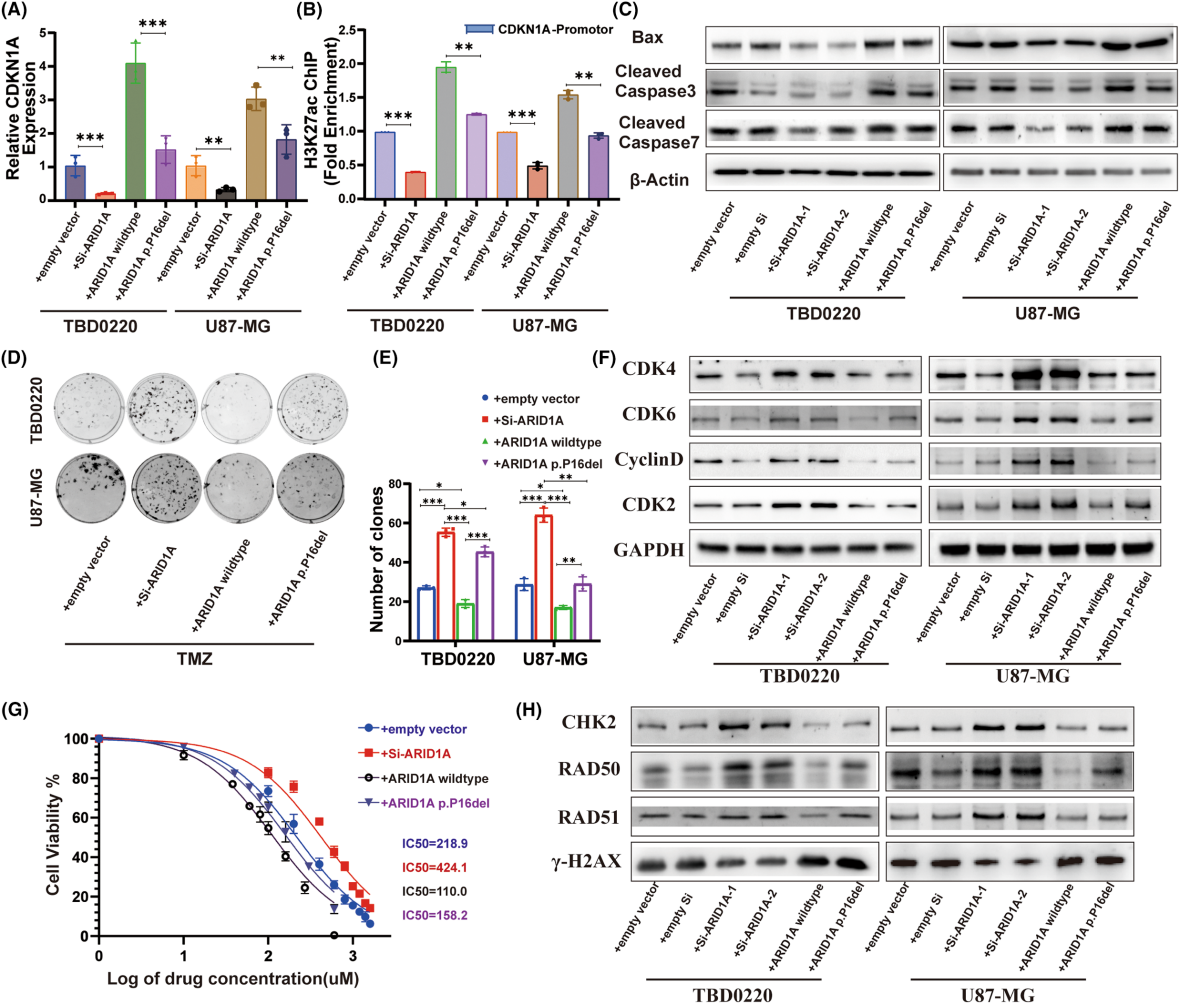
P16 deletion in ARID1A affects DDR pathway after TMZ treatment in glioma cells. (A) qRT‐PCR analysis to determine the differential expression levels of ARID1A and CDKN1A in glioma cells. (B) ChIP was performed in glioma cells using an anti‐H3K27ac antibody against the CDKN1A locus. (C) WB analysis of BAX, Caspase‐3/7, and β‐actin levels in TBD0220 and U87‐MG cell lines. (D) Colony formation assays in GBM cell lines treated with 150 μM of TMZ or DMSO. (E) A colony formation assay was performed in GBM cells. Data are represented as the mean ± SEM (*n* = 3). *p < 0.05, **p < 0.01, ***p < 0.001, ns represents *p* > 0.05. (F) Expression levels of cell cycle‐related proteins CDK4, CDK6, CyclinD, and CDK2 were detected by WB, where GAPDH served as the loading control. (G) CCK8 assays of GBM cell lines treated with TMZ or DMSO. (H) WB analysis of DDR‐related proteins after 200 μM of TMZ exposure.

In this study, we focused on Cell Cycle Protein Dependent Kinase Inhibitor 1A (CDKN1A), which is a regulator of the E2F1 transcription factor and may play a key role in DDR‐related signaling pathways in gliomas and influence TMZ resistance. By qRT‐PCR analysis, we examined the effects of four alterations, ARID1A control, ARID1A KD, ARID1A wild‐type overexpression, and ARID1A‐P16 deletion overexpression, on the expression of CDKN1A, and observed a positive correlation between ARID1A and CDKN1A. Notably, ARID1A‐P16 deletion resulted in decreased CDKN1A expression compared to wild‐type ARID1A overexpression (Figure 5A). The SWI/SNF complex affects histone modification in cells to further explore the effect of ARID1A on glioma cells through histone modification. We investigated the potential mechanism of histone modification of ARID1A with the CDKN1A promoter region using ChIP‐PCR, and showed that ARID1A KD resulted in reduced enrichment of acetylated H3K27 (H3K27ac) in the CDKN1A promoter region. Interestingly, we also observed a reduced level of H3K27ac enrichment in the CDKN1A promoter region of ARID1A‐P16 deletion‐expressing cells compared to wild‐type ARID1A cells (Figure 5B). These findings strongly suggest that ARID1A may affect CDKN1A transcription through histone acetylation modification, which in turn affects the biological activity of glioma cells.

To further understand the effect of ARID1A on apoptosis, we determined the expression of BAX and the downstream effector protein Caspase‐3/7. Our results showed that ARID1A‐P16 deletion attenuated the effect of ARID1A overexpression on apoptosis (Figure 5C). We then performed a CCK8‐based cell viability assay and observed that ARID1A KD resulted in an increase in the IC50 value of TMZ, whereas the IC50 value was decreased upon ARID1A overexpression. Although the TMZ IC50 values of ARID1A‐P16 mutation‐expressing cell lines were decreased, they were still higher than the IC50 values of ARID1A overexpressing glioma cell lines (Figure 5G). In addition, the clonal growth of ARID1A overexpressing cells was significantly lower than that of ARID1A KD cells (Figure 5D,E). To further elucidate the underlying mechanisms, we compared the alterations in cell cycle and DNA damage repair proteins associated with wild‐type ARID1A, ARID1A KD, and ARID1A‐P16 deletion models to those in control glioma lines, following treatment with 200 μM TMZ, using Western blotting. We found that the expression levels of CDK4, CDK6, Cyclin D1, and CDK2 were significantly decreased in wild‐type or P16 deletion variant cells transfected with ARID1A compared to controls, but the expression levels of CDK4, CDK6, Cyclin D1, and CDK2 were higher in cells transfected with the P16 deletion plasmid than in cells transfected with wild‐type plasmid (Figure 5F). After TMZ treatment, we found that DDR proteins including RAD50 were also affected (Figure 5H). These results suggest that P16 deletion in ARID1A can regulate the stability of the SWI/SNF complex and further promote the resistance of glioma cells to TMZ in vitro.

### 2.6 ARID1A KD increases GBM resistance to TMZ in vivo

**FIGURE 6:**
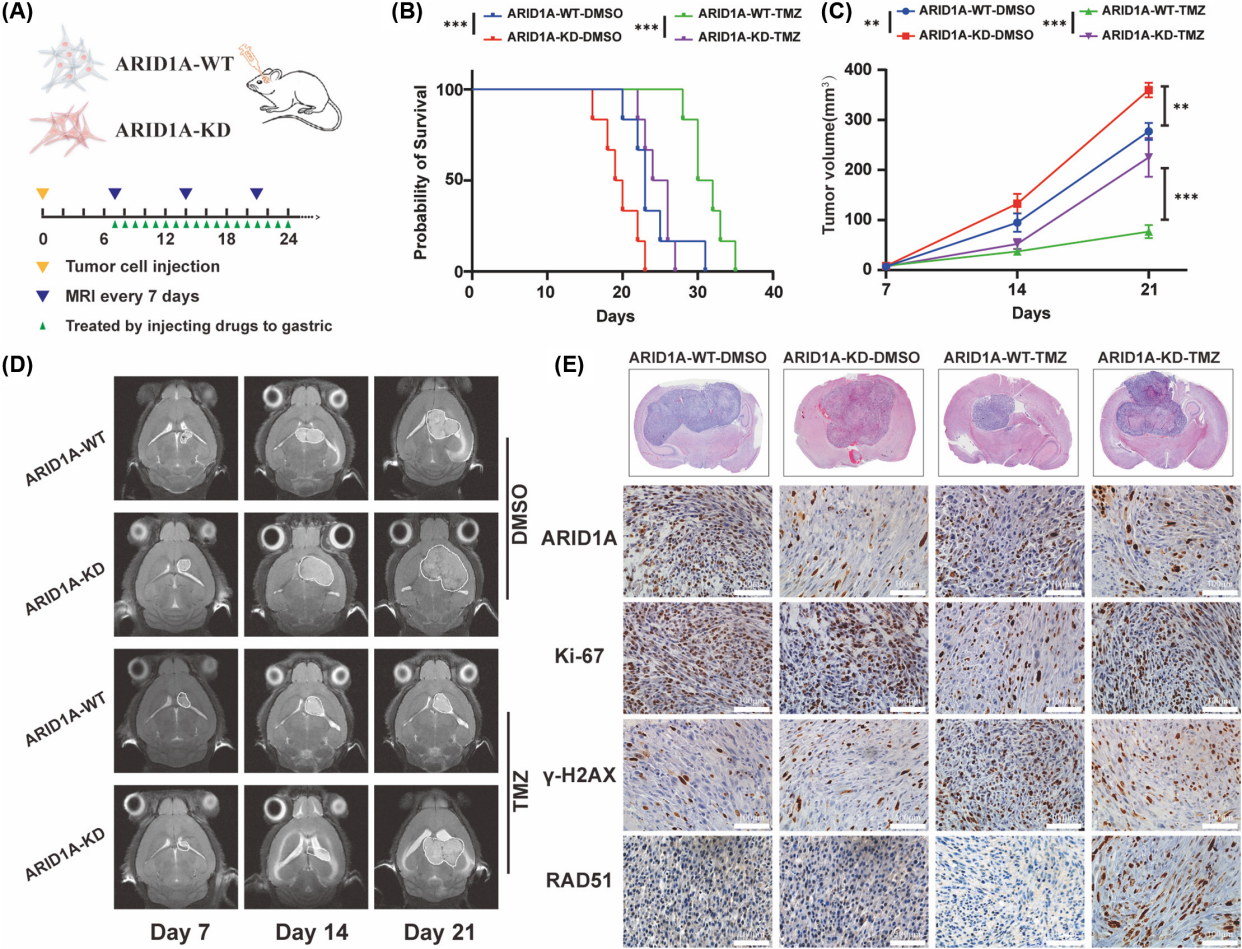
ARID1A KD promotes GBM proliferation and reduces survival time in tumor‐bearing mice. (A) The flow diagram of nude mice xenograft model. (B) Kaplan–Meier curves for different experimental and control groups. *p < 0.05, **p < 0.01, ***p < 0.001 (Log‐rank test). (C) Tumor volumes were measured by magnetic resonance imaging (MRI) of the brain on days 7, 14, and 21 after implantation, tumor growth curves were quantitated and illustrated. *p < 0.05, **p < 0.01, ***p < 0.001 (two‐way ANOVA). (D) MRI imaging of representative groups of mice. (E) Representative images of formalin‐fixed and paraffin‐embedded (FFPE) brain tissues for H&E and IHC staining of Ki67, RAD51, and γ‐H2AX. Scale bar = 100 μm.

Having studied the effect of ARID1A mutation on glioma in clinical tumor samples and at the in vitro cytological level, we further investigated the effect in experimental animals. We established an in situ xenograft glioma mouse model by transplanting TBD0220 cells into 5‐week‐old female BALB/c nude mice (Figure 6A), which were divided into a control group and an ARID1A KD group, and underwent gavage treatment with TMZ after randomization to a randomized group on day 7 postoperatively to mimic the process of chemotherapy in clinical patients, respectively. Magnetic resonance imaging (MRI) was performed on days 7, 15, and 21 postoperatively to observe the growth of tumors in each group and to record the survival curves and the growth status of the mice. The results showed that ARID1A KD effectively promoted tumor growth with more malignant biological characteristics, whereas TMZ treatment was not effective in inhibiting tumor proliferation of gliomas in ARID1A KD mice (Figure 6C,D). Kaplan–Meier survival curve analysis showed that the median survival of ARID1A KD GBM mice was significantly shorter, whereas TMZ treatment ARID1A KD mice had a further shortened median survival of 25 days compared to 31 days for control mice (Figure 6B). The appropriate time for euthanasia was selected according to the tumor size and the physical condition of the mice. Animal specimens were taken and subjected to a series of experiments, and H&E staining of the specimens confirmed that the tumor volume of the ARID1A KD group was significantly larger than that of the control group, and IHC analysis showed that Ki‐67 expression was higher in the ARID1A KD group than that of the control group. In addition, increased levels of the DDR factor RAD51 and decreased levels of γH2AX were observed in tumor sections from the TMZ treatment group (Figure 6E). These results suggest that ARID1A KD leads to a significant increase in the TMZ resistance profile of GBM cells, as evidenced by reduced tumor regression and increased tumor volume in the KD group relative to the control group. These findings highlight the critical role of ARID1A in modulating the response of GBM tumors to TMZ and underline the potential clinical significance of targeting ARID1A in GBM therapy.

## 3 MATERIALS AND METHODS

### 3.1 Patient cohorts

We enrolled glioma patients who underwent surgery at the Affiliated Hospital of Hebei University from October 1, 2018, to December 31, 2020. The recruited patients met the following selection criteria: (1) patients with primary glioma had not received any prior adjuvant chemotherapy or radiotherapy before surgery and (2) no evidence of distant metastasis at the time of diagnosis in enrolled patients. H&E‐stained sections were available for all cases in this cohort. Clinical follow‐up data for the dataset cohort was available in the dataset cohort of follow‐up patients until September 1, 2021, with a median follow‐up of 803 days. Overall survival (OS) was defined as the time from diagnosis to death from any cause. Patients with PFS were censored from the time point of the last follow‐up visit. All collected cancer tissues were validated by experienced pathologists by H&E analysis. Somatic cell samples paired with tumor tissues were collected before the surgery, immediately snap‐frozen in liquid nitrogen and stored at −80°C until genetic information was extracted. Written informed consent was obtained from each patient. The study was approved by the Institutional Review Board (IRB) of the Affiliated Hospital of Hebei University.

### 3.2 Targeted deep sequencing

Genomic DNA was extracted from matched tumor tissue and somatic cell samples using the QIAamp DNA kit (QIAGEN, Hilden, Germany). Then sequencing libraries were constructed, purified, quantified, and validated. A total of 428 candidate genes (Table S2) were deeply sequenced using the NovaSeq6000 sequencing platform with PE150 strategy. We used muTect and Strelka for somatic SNV analysis in parallel and ANNOVAR for functional annotation of SNVs. Strelka was used for somatic INDEL analysis, and ANNOVAR for functional annotation of INDEL. Raw Illumina reads of whole genome sequencing (WGS) were processed for quality control using FastQC. We used the R version 4.0.5 maftools package^10^ to visualize mutations in sequencing results and to analyze the reciprocity of variables.

### 3.3 Public database analysis

Public data were downloaded from the publicly available TCGA and CGGA databases. Kaplan–Meier survival analysis curves were plotted using the ggsurvplot package of the R version 4.0.5.

### 3.4 Cell culture

Primary patient‐derived GBM cell line TBD0220 was cultured in Dulbecco's modified Eagle medium (DMEM/F12, 1:1; Gibco) supplemented with 10% fetal bovine serum (FBS). The U87‐MG cell line was purchased from American Type Culture Collection (ATCC) and cultured in DMEM containing 10% FBS. All cells were grown in a humidified incubator at 37°C with 5% CO_2_.

### 3.5 Cell transfection

Lipofectamine™ 3000 transfection reagent (Invitrogen™) was used for transfecting siRNA and expression plasmids into 70%–80% confluence glioma cells, according to the manufacturer's instructions, and in vivo experiments were performed using lentiviral ARID1A shRNA (ARID1A‐KD) plasmid. Transfected virus‐positive cells were selected with puromycin (2 μg/mL) for 2 weeks to generate a stable shRNA expression cell model. The transfection efficiency of ARID1A plasmid was confirmed by qRT‐PCR and WB. All siRNA sequences were synthesized by IBSBio (Shanghai, China) (Table S3).

### 3.6 RNA extraction and qRT‐PCR

Total RNA was extracted using the TRIzol reagent (15596‐026, Thermo Fisher), and cDNA was synthesized using the Prime‐Script RT reagent kit (Takara). cDNA levels were quantified using SYBR Green reaction mix (Takara) in a QuantStudio 3 Real‐Time PCR system (Thermo‐Fisher Scientific). Individual samples were loaded in triplicates. GAPDH was used as an internal reference. The relative expression of mRNA was quantified by the 2^−ΔΔCt^ method. Primer sequences used for qRT‐PCR assays are listed in Table S4.

### 3.7 Western blot analysis

Briefly, after performing the indicated treatments, all proteins were extracted with RIPA buffer (Solarbio, Beijing, China), and protein quantification was performed with a BCA assay kit (Solarbio, Beijing, China). Protein lysates were separated by SDS–polyacrylamide gel electrophoresis (PAGE) electrophoresis and then transferred onto polyvinylidene fluoride (PVDF) membranes (Millipore, USA). PVDF membranes were incubated overnight at 4°C with rabbit anti‐ARID1A (1:1000, 12354S, CST), rabbit anti‐CDK2 (1:10,000, ab32147, Abcam), rabbit anti‐CDK4 (1:1000, DF6102, Affinity), rabbit anti‐CDK6 (1:100,000, ab124821, Abcam), rabbit anti‐Cyclin D (1:2000, AF0931, Affinity), mouse anti‐GAPDH (1:50,000, 60004‐1‐Ig, Proteintech), rabbit anti‐BAX (1:1000, 41162S, CST), rabbit anti‐Cleaved Caspase‐3 (1:1000, 9664S, CST), rabbit anti‐Cleaved Caspase‐7 (1:1000, 8438S, CST), rabbit anti‐β‐actin (1:10,000, AF7018, Affinity), rabbit anti‐RAD50 (1:5000, 29390‐1‐AP, Proteintech), rabbit anti‐CHK2 (1:1000, 6334S, CST), rabbit anti‐RAD51 (1:2000, 14961‐1‐AP, Proteintech), rabbit anti‐γ‐H2AX (1:1000, ab229914, Abcam), rabbit anti‐BRG1 (1:1000, 49360S, CST), followed by a 1 h incubation at room temperature with the horseradish peroxidase (HRP)‐conjugated respective secondary antibody for chemiluminescence‐based protein detection.

### 3.8 Cell viability, clonogenic assays

For the cell viability assay, 2 × 10^3^ cells per well were seeded overnight in 96‐well plates before drug treatment. Cell Counting Kit‐8 (CCK‐8) assay (Dojindo) was used to assess cell viability at indicated time points. Clone formation assay was performed by seeding approximately 300 cells per well in 6‐well plates and incubating them in an incubator for 14 days after the indicated treatment, followed by fixation with 4% paraformaldehyde (PFA). Then, the number of clones was counted after staining with crystal violet.

### 3.9 Flow cytometry analysis

Cell cycle status was detected using the Cell Cycle and Apoptosis Analysis Kit (Beyotime). FITC Annexin‐V and 7‐AAD (BD Pharmingen) were used to detect the population of apoptotic cells in each group by flow cytometry (BD FACSCanto II).

### 3.10 Co‐immunoprecipitation (Co‐IP)

Cell lysates were prepared using NP‐40 lysate (Beyotime), and added with PMSF. After protein quantification using BCA assay kit (Solarbio), rabbit anti‐His‐Tag (1:25, 2365, CST) antibody was added to 200 μL of cell lysates at 1 mg/mL concentration and incubated overnight at 4°C. After incubation with 40 μL of protein‐A/G magnetic beads (Bimake) for 3 h, the precipitates were washed five times with IP lysis buffer and the target proteins were eluted and detected by WB.

### 3.11 Chromatin immunoprecipitation (ChIP)

ChIP assays were performed using the Magna ChIP™ A/G Chromatin Immunoprecipitation kit. The purified DNA samples were quantified by qRT‐PCR. The primer sequences used are listed in Table S4.

### 3.12 Confocal immunofluorescence (IF) microscopy

For IF, cells were treated as indicated for 24 h. After the cells were fixed with 4% PFA diluted in 1 × PBS for 15 min at room temperature, the fixative was aspirated, and cells were rinsed with 1 × PBS rinsed three times for 5 min each. Then, cells were treated with 0.5% Triton‐X100 (Thermo‐Fisher) diluted in warm PBS and blocked with blocking buffer (5% bovine serum albumin (BSA) diluted in warm PBS, BioFroxx, Guangzhou, China) for 1 h at room temperature. After that, cells were incubated with the target primary antibody overnight at 4°C, followed by the secondary antibody for 1 h at 37°C. Nuclear staining was performed using 1 μg/mL of 4′‐6‐diamidino‐2‐phenylindole (DAPI, Molecular Probes, D1306). Protein subcellular localization was then observed with a confocal laser scanning microscope (CLSM, Zeiss 510 META).

### 3.13 Hematoxylin and eosin (H&E) staining and immunohistochemistry (IHC)

Brain sections were cut from paraffin‐embedded brain tissue blocks. Paraffin‐embedded tissue sections were used for H&E staining. For IHC, brain sections were dewaxed, hydrated, treated in 1 × EDTA buffer (Zsbio) at 100°C for 20 min for antigen retrieval, then incubated with goat serum at room temperature for 30 min, followed by overnight incubation at 4°C with rabbit anti‐ARID1A (1:2000, 12354S, CST), rabbit anti‐Ki67 (ZM‐0167, ZSGB‐BIO), rabbit anti‐p‐ERK1\2 (1:300, 4370S, CST), rabbit anti‐RAD51 (1:500, 14961‐1‐AP, Proteintech), rabbit anti‐γ‐H2AX (1:200, ab229914, Abcam), The next day, slides were rinsed with 1 × PBS three times, followed by incubation with enzyme‐labeled secondary antibody at room temperature for 1 h. Color development was performed using diaminobenzidine (DAB) reagent, followed by image acquisition under brightfield microscopy.

### 3.14 In vivo xenograft mouse models

Animal experiments were performed according to the animal study protocols approved by the Hebei University Institutional Animal Care and Use Committee. The GBM xenograft model was constructed in 4‐week‐old BALB/c nude mice. Four groups (*n* = 10 per group) of mice were randomly selected, and TBD0220 cells (1 × 105 cells per mouse in 3 μL PBS) were injected intracranially under the guidance of a stereotactic apparatus with coordinates relative to bregma: 2.0 mm posterior, 2.0 mm lateral, and 3.0 mm ventral to establish the GBM model. The intracranial tumors were measured with Bruker 9.4T BioSpec 94/30 MRI & PET Insert, and the consecutive sections (0.5 mm) were obtained on the 7th, 15th, and 21st day after tumor implantation. In addition, mice were monitored for survival during tumor progression, and OS curves were generated using the Kaplan–Meier method. After death or euthanasia, brain tissues were harvested, fixed in 4% PFA for 24 h, paraffin‐embedded, and sectioned into 5 μm slices for IHC and H&E staining.

### 3.15 Statistical analysis

Statistical analyses were performed using GraphPad Prism 8.4.0 software (https://www.graphpad.com) and SPSS 22.0 software. Gene enrichment analysis was performed using metascape.^11^ All data were tested for normality using the Shapiro–Wilk test for non‐parametric tests in SPSS. The student's *t*‐test was used to compare two experimental groups, and one‐way or two‐way ANOVA was used to compare three or more experimental groups, and the error bars in the graphs represent the mean ± standard deviation (SD). *p* < 0.05 was considered statistically significant. All results were repeated at least three independent times.

### 3.16 Data and code availability

All sequencing and metabolomics data in this study are available from the Lead Contact, Chuan Fang (chuanfang@hbu.edu.cn), upon reasonable request.

## 4 DISCUSSION

In this study, deep targeted sequencing was employed to examine the variance in mutation frequency of the ARID1A gene between primary glioblastoma and non‐glioblastoma, utilizing mismatch repair genes and tumor‐frequent mutation genes as benchmarks. The resultant simulations and protein interaction analyses disclosed that the deletion of proline at position 16 in the ARID1A protein compromises the stability of its binding to the SWI/SNF core subunit BRG1, which, in turn, influences the SWI/SNF complex's activity. This event gives rise to modified histone modifications in the CDKN1A promoter region, which subsequently impacts the biological activity of glioma cells.

By scrutinizing exome sequencing results from The Cancer Genome Atlas (TCGA) and CGGA, we identified a range of potential driver genes, encompassing well‐known genes such as EGFR, TP53, IDH1, and CDKN2A, alongside novel genes LZTR1 and the FGFR‐TACC fusion.^12^, ^13^, ^14^ Upon integrating deep targeted sequencing data, we observed that the ARID1A gene was significantly more frequently mutated in WHO grade 4 gliomas than in WHO grades 1–3 gliomas, a phenomenon that ignited our research curiosity.

ARID1A (AT‐rich interactive domain‐containing protein 1A) is an epigenetic regulator, a component of the SWI/SNF complex, a key player in epigenetic regulation responsible for remodeling chromatin structure and influencing gene expression.^15^, ^16^ In cancer, dysfunctional SWI/SNF complexes are intimately associated with tumor development and progression.^17^ In gliomas, ARID1A mutations in WHO grade 3 oligodendrocyte tumors are linked to poorer progression‐free survival,^18^ yet the role of ARID1A in GBM remains to be fully unraveled.

In our study, mutations in ARID1A that disrupt its function were predominantly observed in glioblastoma multiforme (GBM). Moreover, the decreased expression of ARID1A, as noted in the TCGA database, correlated with poorer survival outcomes. Additionally, the concomitant reduction in ARID1A expression and mutations in NF1 was linked to an unfavorable prognosis, a finding substantiated by immunohistochemical analysis of clinical specimens. NF1 is a RasGTPase‐activating protein, which enhances the GTP hydrolysis activity of Ras, thereby attenuating Ras signaling.^19^ Mutations in NF1 result in the hyperactivation of the Ras pathway, which in turn leads to increased expression of genes involved in cell proliferation and survival, while downregulating genes that inhibit the cell cycle. Such dysregulation may further impair the normal function of the SWI/SNF complex. The ensuing dysfunction of the SWI/SNF complex can diminish the cellular response to tumor suppressor signals, thereby facilitating tumor progression.

Additionally, alterations in ARID1A interact with the PI3K/Akt/mTor pathway in nasopharyngeal carcinoma and gastric cancer cell lines.^20^, ^21^ We further postulated that ARID1A deletion as a component of the SWI/SNF complex can disrupt the complex's assembly, which operates by harnessing the ATPase subunit's energy to remodel the nucleosome, thereby permitting chromatin access to transcription factors and consequently regulating gene expression.^22^ This illuminates the aforementioned phenomenon.

Next, the study modeled the structure of the protein encoded by the gene locus with the high‐frequency mutation and found that the deletion of the amino acid at position 16 of ARID1A impairs its binding to the SWI/SNF complex's core subunit BRG1, leading to the abnormal function of the complex. Hence, by starting with the SWI/SNF complex's function and searching for its regulated transcription factors, certain findings substantiated that the E2F family proteins in gliomas play a pivotal role in DNA damage repair and sustaining cell survival, leading to TMZ (temozolomide) resistance.^23^ Our results affirmed a correlation between its expression and ARID1A. Further, by assessing changes in histone modifications in the CDKN1A promoter region, which regulates the E2F1 transcription factor, we determined that ARID1A deletion downregulates CDKN1A acetylation, diminishes P21 protein transcription, and initiates a cascade of tumor drug resistance responses.

In summary, our results propose that ARID1A not only acts as a predictive biomarker in gliomas but also that its deletion, by undermining the stability of the SWI/SNF complex, may cause gliomas to exhibit more malignant features. Changes in the SWI/SNF complex's stability, in turn, affect histone modifications in the glioma cells' CDKN1A promoter region, leading to TMZ drug resistance. Through deep targeted sequencing to detect mutations in tumors, and evaluating the significance of these mutations, we aim to forecast disease prognosis and forge more personalized therapeutic strategies, providing a logical pathway for glioma treatment. These findings offer new insights into ARID1A's function and mechanism, paving new avenues for glioma therapy. By targeting the regulation of ARID1A and SWI/SNF complexes, it may be feasible to develop more effective therapeutic approaches to enhance the survival and quality of life of glioma patients. Our findings hold substantial potential value for clinical applications and are poised to contribute to the realization of personalized medicine and precision therapy.

## AUTHOR CONTRIBUTIONS

Chuan Fang, Chunsheng Kang, and Jie Li conceptualized and designed the study. Can Xu, Jixing Zhao, Shixue Yang, Biao Hong conducted the experiments. Yanli Tan, Jie Zhang, Xiang Li provided support with managing GBM clinical samples. Menglin Xiao and Xiaoteng Cui analyzed the data, generated the figures, and prepared the manuscript. All authors approved the final version of the manuscript.

## CONFLICT OF INTEREST STATEMENT

The authors declare no conflicts of interest.

## Supporting information


Figures S1–S4

Table S1

Table S2

Table S3

Table S4

Data S1

## Data Availability

The data that support the findings of this study are available on request from the corresponding author. The data are not publicly available due to privacy or ethical restrictions.
