# Supplementary material for: Deep‐targeted gene sequencing reveals ARID1A mutation as an important driver of glioblastoma
Source: CNS Neurosci Ther. 2024 Apr 11;30(4):e14698. doi: 10.1111/cns.14698 (PMC11007544; doi:10.1111/cns.14698)
Supplement: Supplementary file 1 — Figures S1–S4 [file CNS-30-e14698-s002.zip › Supplementary Figures.docx]

**Supplementary information**

**Deep targeted gene sequencing reveals ARID1A mutation as an important driver of glioblastoma**

Menglin Xiao, Xiaoteng Cui, Can Xu, Lei Xin, Jixing Zhao, Shixue Yang, Biao Hong, Yanli Tan, Jie Zhang, Xiang Li, Jie Li, Chunsheng Kang, Chuan Fang


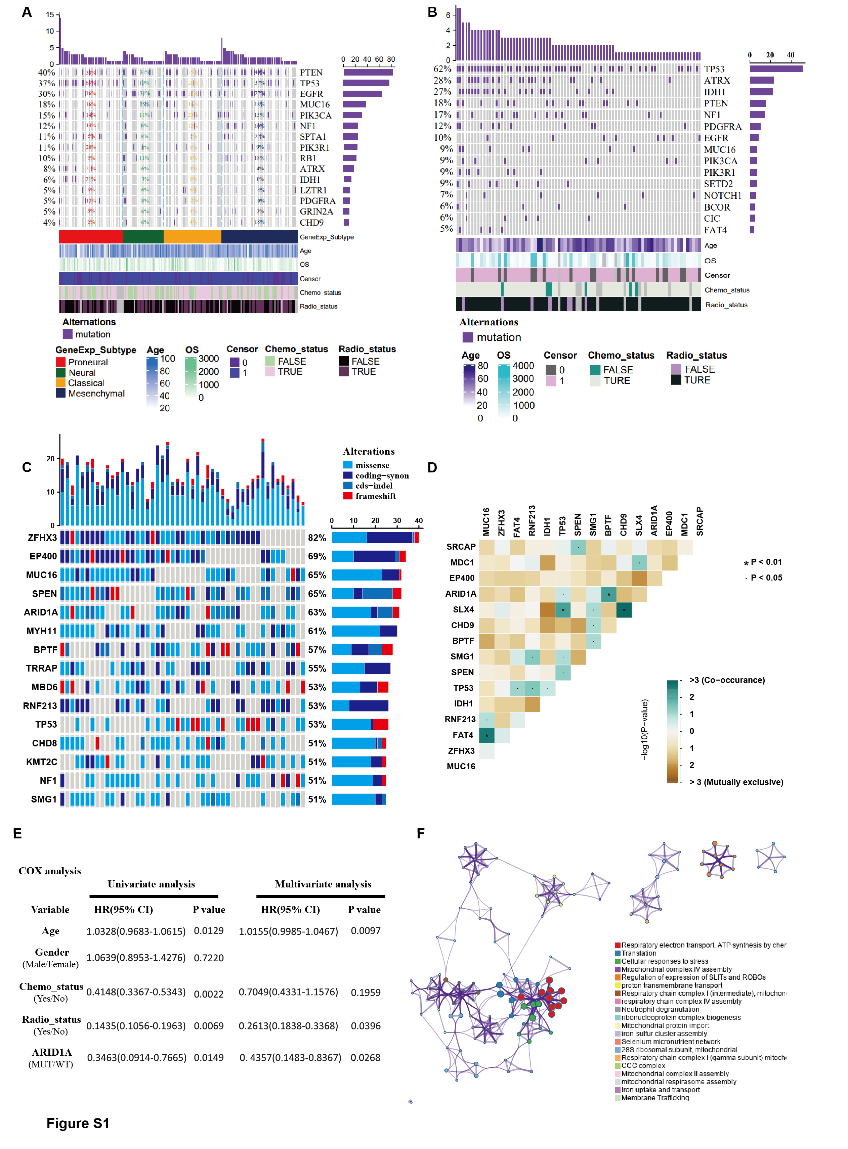
**Figure S1.**

1. Waterfall plot of the 15 genes with the highest mutation frequency in the TCGA database GBM dataset.
2. Waterfall plot of the 15 genes with the highest mutation frequency in the CGGA database GBM dataset.
3. Waterfall plot of the 15 genes with the highest mutation frequency in the WHO grade 4 glioma validation cohort
4. Mutual exclusion and co-occurrence of GBM top15 genes in the dataset cohort.
5. Univariate and multivariate analyses of the ARID1A and other clinical information in relation to the overall survival in the dataset cohort.
6. GO and KEGG pathway analysis of genes negatively associated (R<-0.6) with ARID1A in the TCGA database GBM dataset.


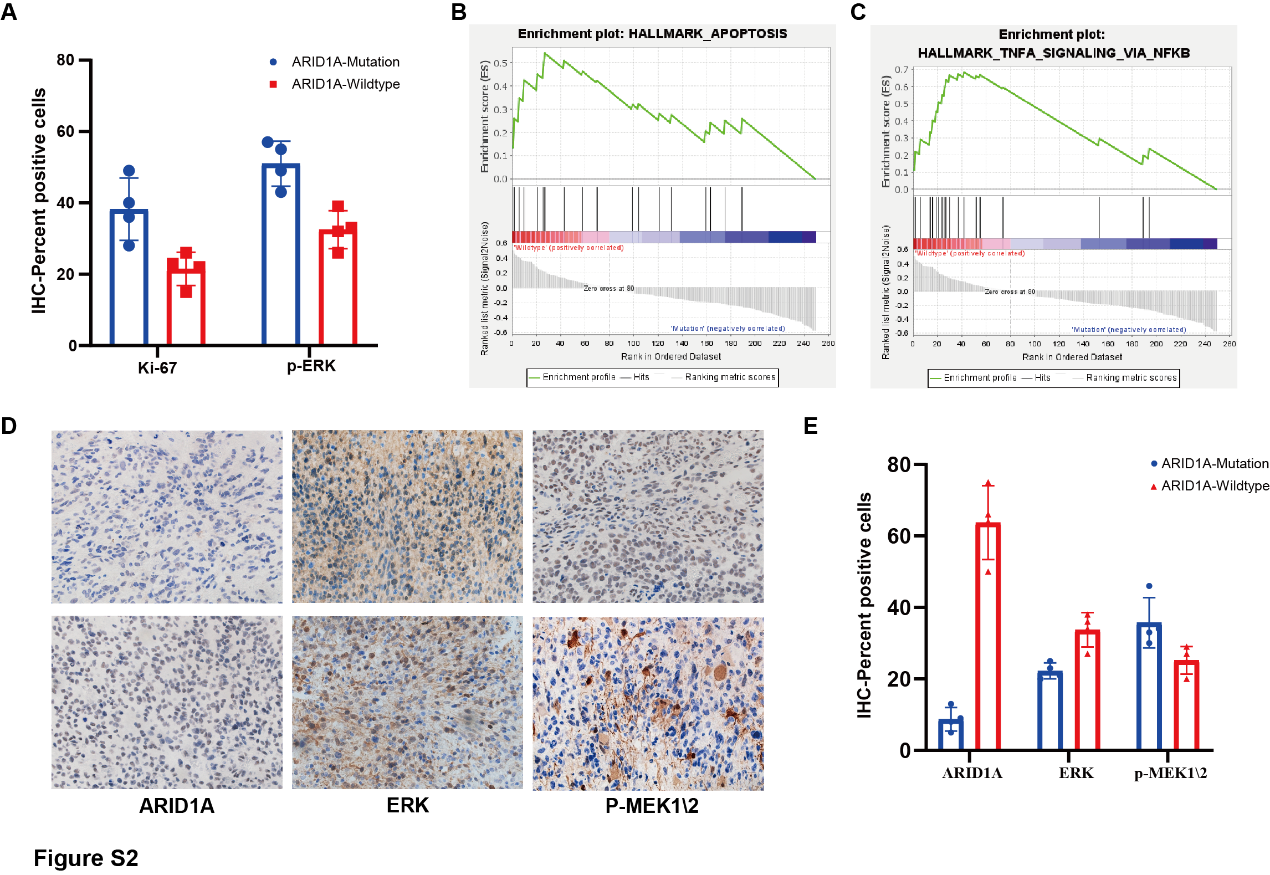


**Figure S2.**

(**A**) Statistical analysis of clinical specimens stained with IHC for Ki-67, p-ERK

(**B&C**) GSEA enrichment analysis of wild-type and mutant ARID1A sample RAF signaling pathway genes in the CGGA database.

(**D**) Representative images of paraffin-embedded (FFPE) clinical specimens subjected to IHC staining for ARID1A, ERK, and p-MEK1/2.

(**E**) Statistical analysis of clinical specimens stained with IHC for ARID1A, ERK, and p-MEK1/2


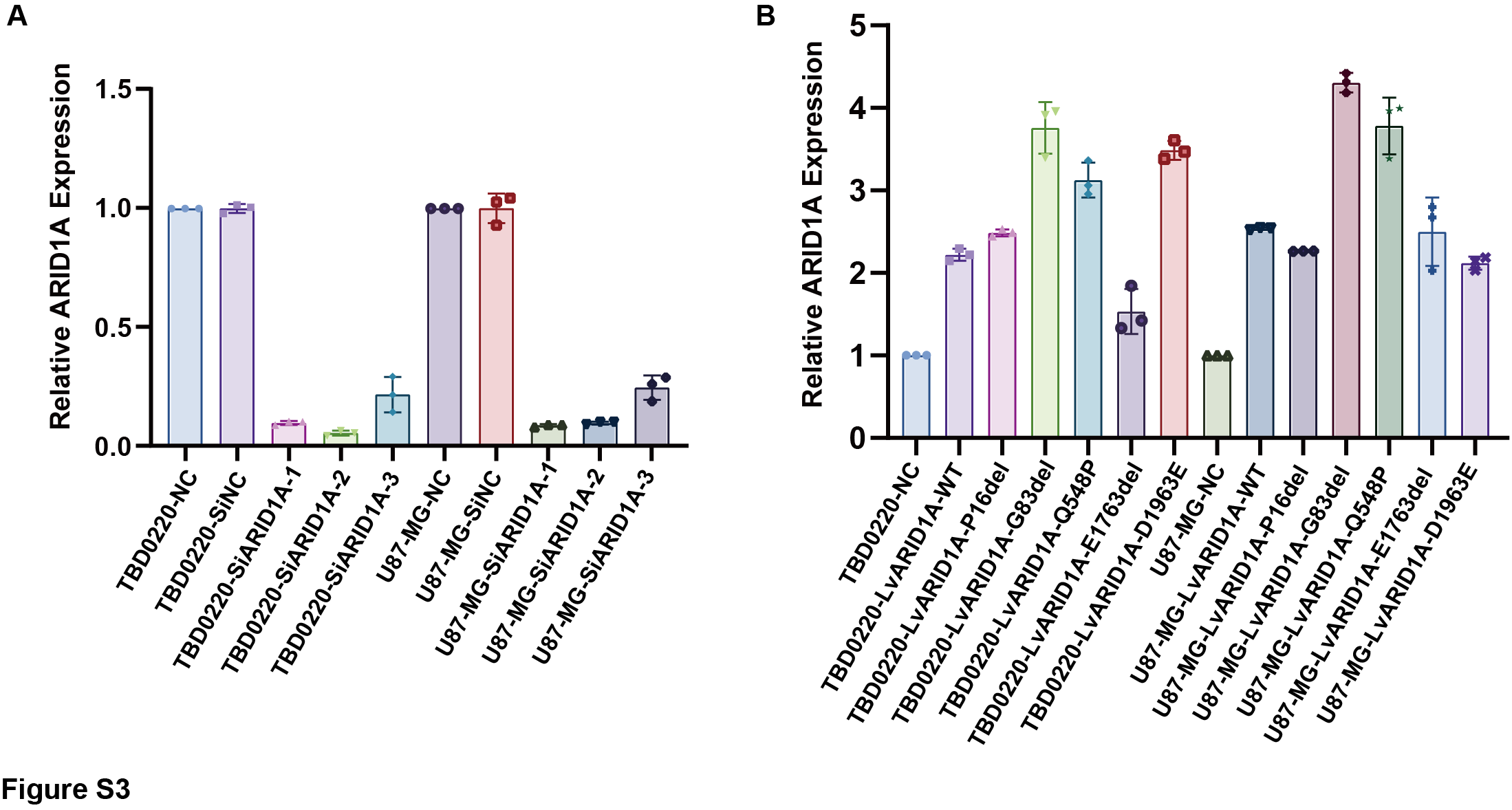


**Figure S3.**

(**A**) qRT-PCR analysis to determine the efficiency of SiRNA in knocking down ARID1A in glioma cells.

(**B**) qRT-PCR analysis to determine the efficiency of mutants in glioma cells.


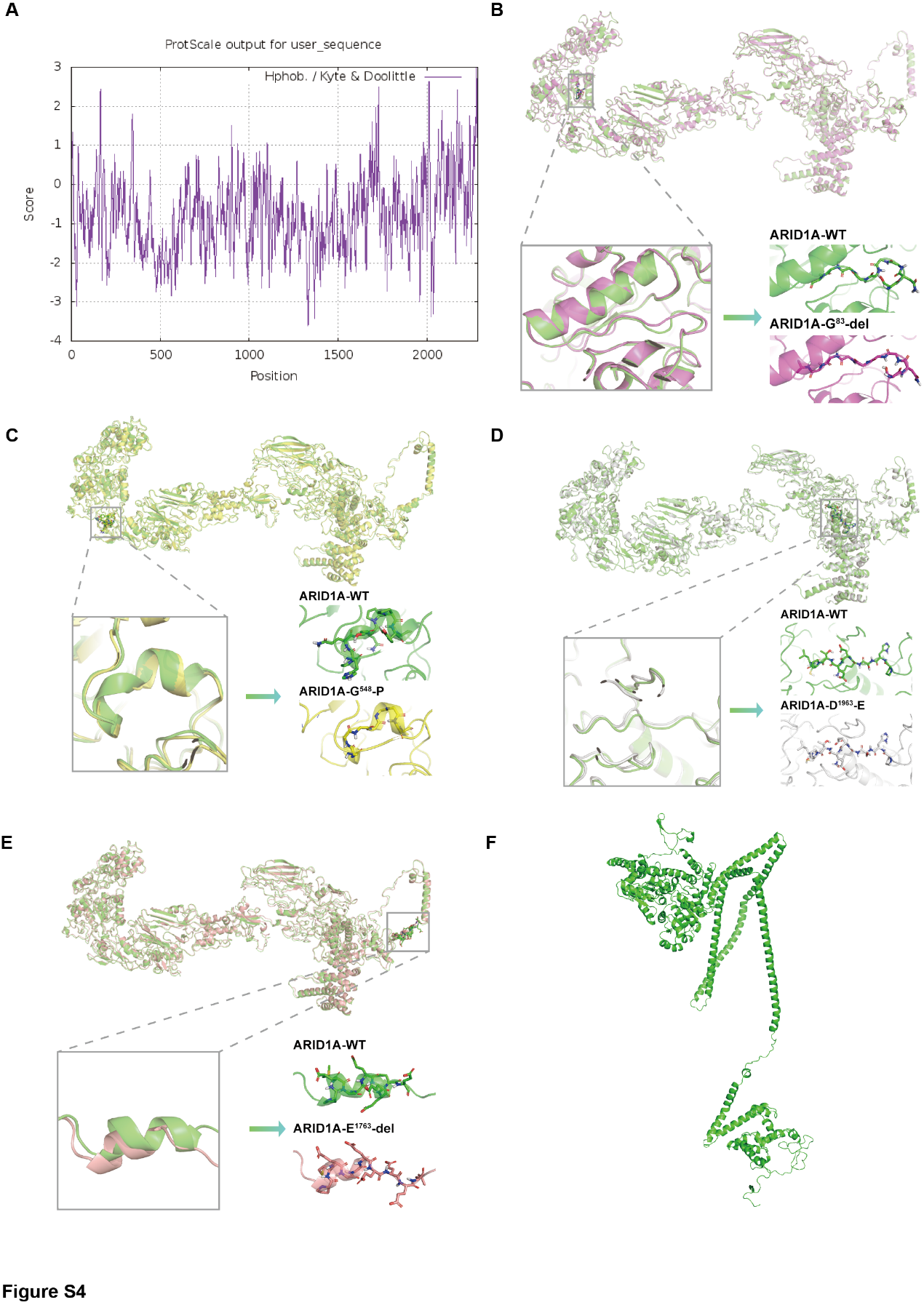
**Figure S4.**

1. Hydrophobic analysis of the content of hydrophilic residues in ARID1A.

(**B-E**) Simulated conformational changes of the ARID1A high-frequency mutation.

(**F**) 3D structure of BRG1 after optimization by GROMACS 2018 kinetic software.
