# Supplementary figures and images for: Deep‐targeted gene sequencing reveals ARID1A mutation as an important driver of glioblastoma
Source: CNS Neurosci Ther. 2024 Apr 11;30(4):e14698. doi: 10.1111/cns.14698 (PMC11007544; doi:10.1111/cns.14698)

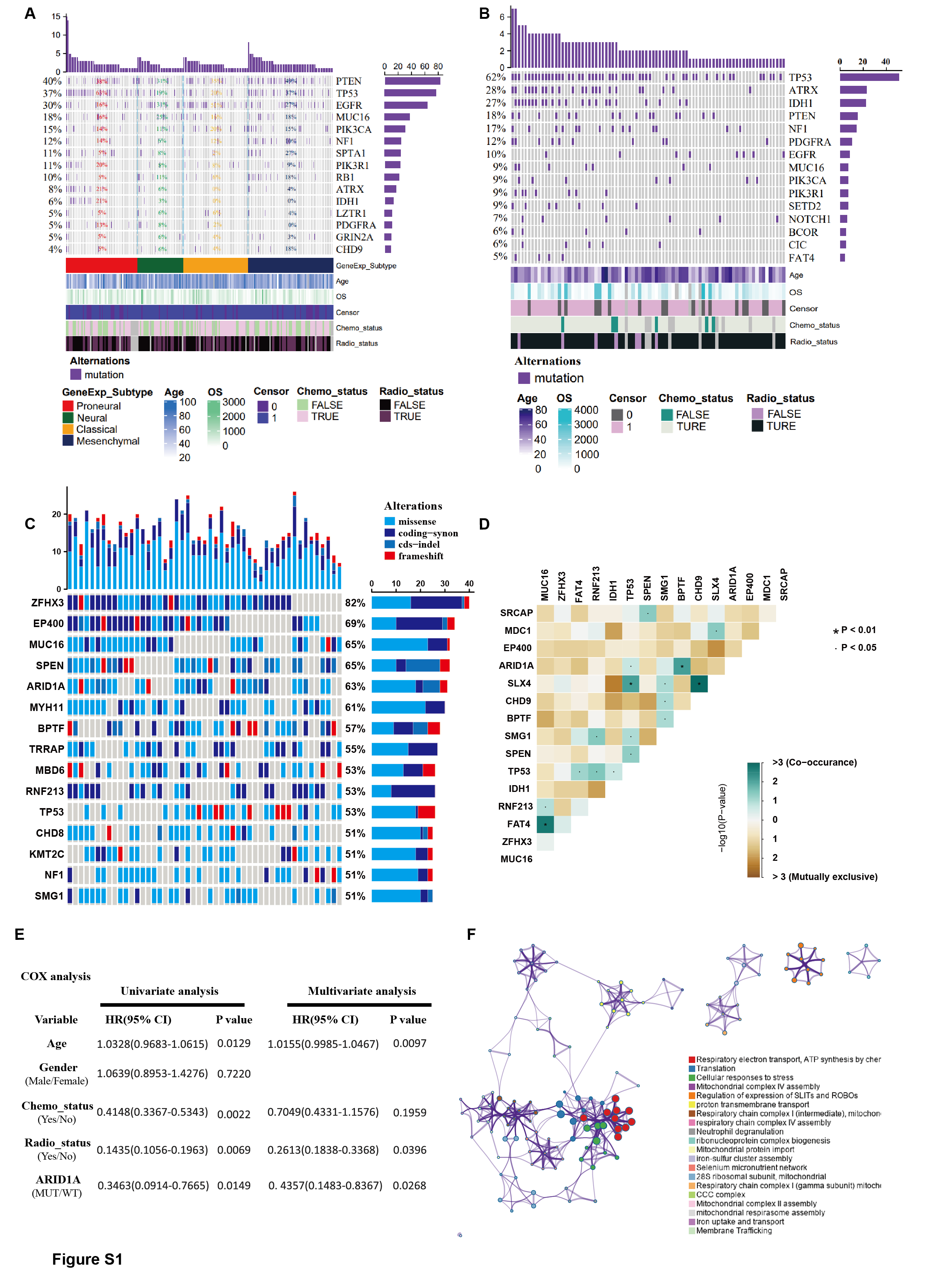

Supplement: Supplementary file 1 — Figures S1–S4 [file CNS-30-e14698-s002.zip › Supplementary Figure 1.png]

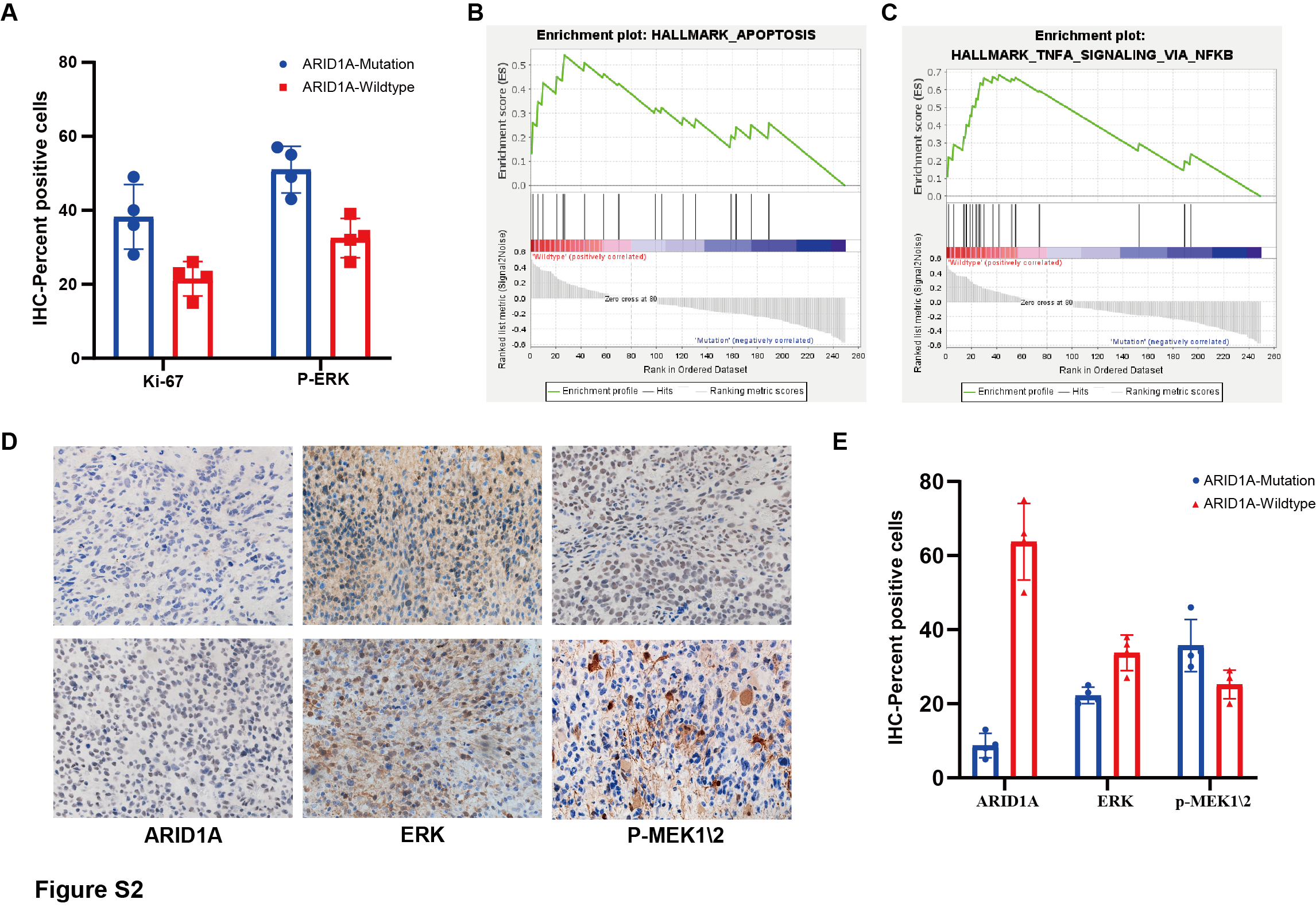

Supplement: Supplementary file 1 — Figures S1–S4 [file CNS-30-e14698-s002.zip › Supplementary Figure 2.png]

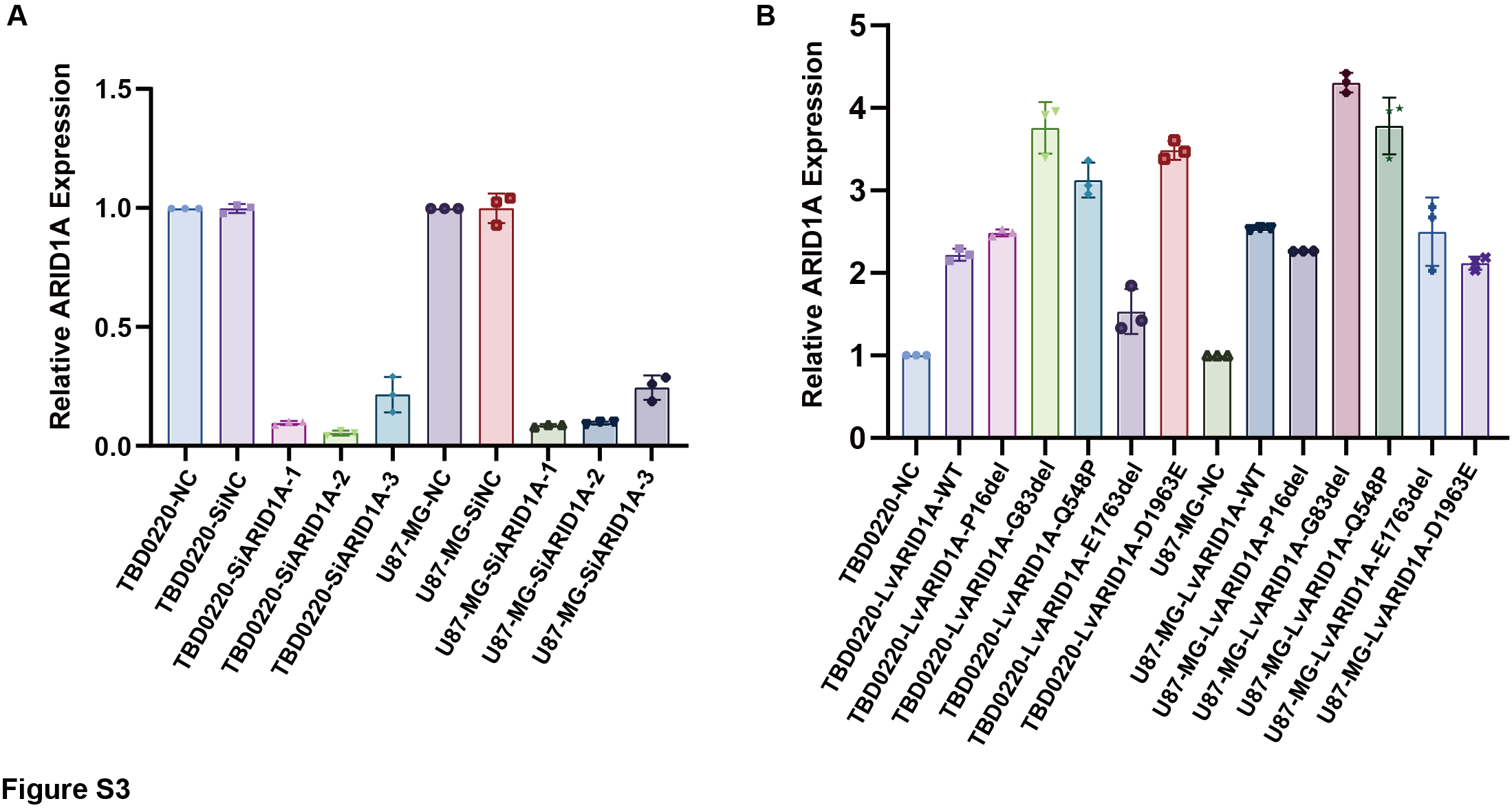

Supplement: Supplementary file 1 — Figures S1–S4 [file CNS-30-e14698-s002.zip › Supplementary Figure 3.png]

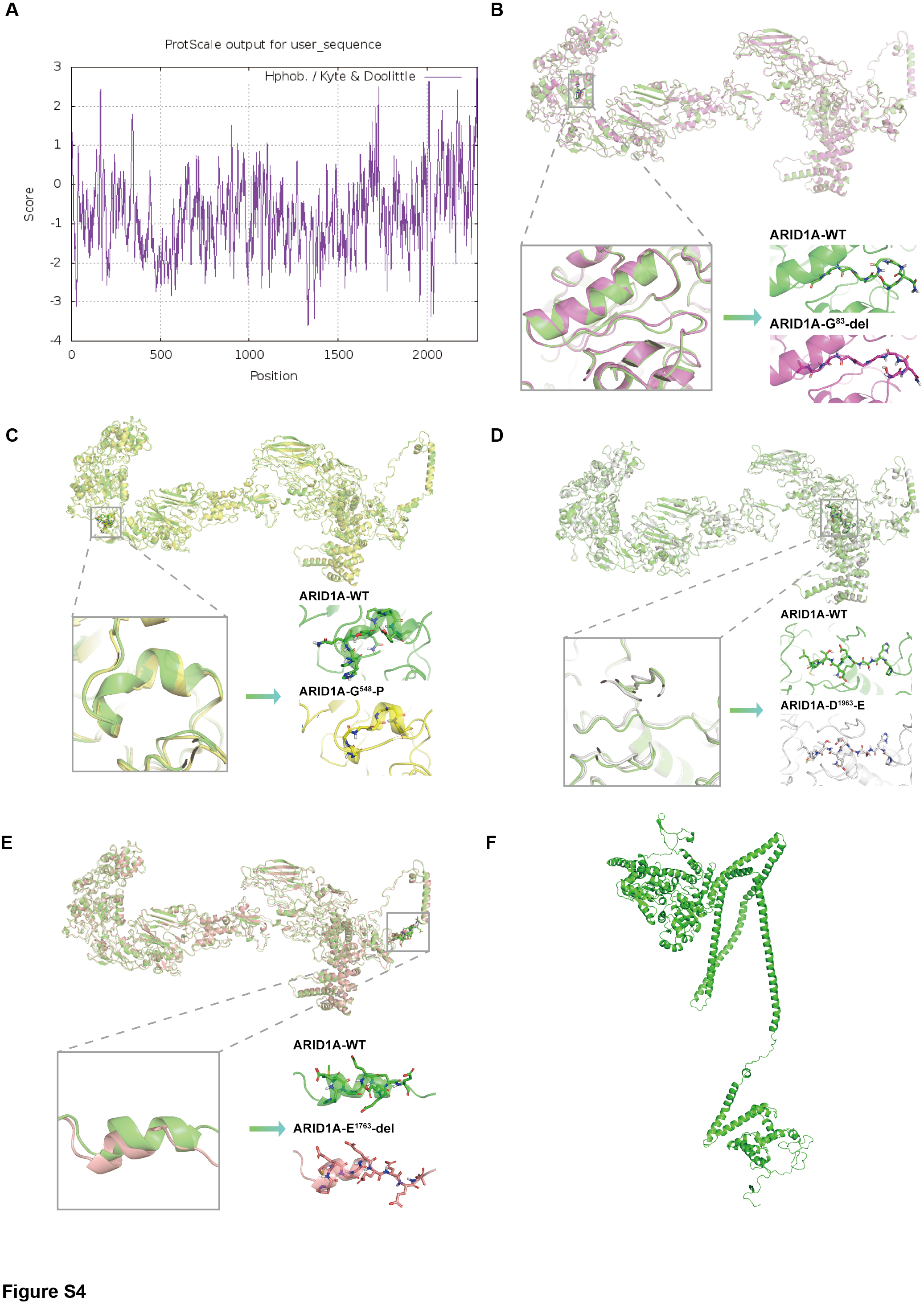

Supplement: Supplementary file 1 — Figures S1–S4 [file CNS-30-e14698-s002.zip › Supplementary Figure 4.png]
