# Supplementary material for: Deep‐targeted gene sequencing reveals ARID1A mutation as an important driver of glioblastoma
Source: CNS Neurosci Ther. 2024 Apr 11;30(4):e14698. doi: 10.1111/cns.14698 (PMC11007544; doi:10.1111/cns.14698)
Supplement: Supplementary file 2 — Table S1 [file CNS-30-e14698-s006.docx]

Table S1 :Results of BRG1 comparison with high homology template sequences respectively

| RANK | PDB HIT | Length | Identity | Cover | Max score |
| --- | --- | --- | --- | --- | --- |
| 1 | 7VDV_A | 1485 | 97.21% | 89% | 2170 |
| 2 | 6UXV_A | 1703 | 53.60% | 50 | 708 |
| 3 | 5HZR_A | 732 | 55.42% | 43 | 706 |
| 4 | 7EGM_A | 982 | 53.60% | 44 | 704 |
| 5 | 5X0X_O | 735 | 53.60% | 40 | 704 |
| 6 | 6IY2_O | 679 | 61.48% | 31 | 682 |
| 7 | 6K15_J | 1359 | 62.36% | 49 | 652 |
| 8 | 6VZ4_K | 813 | 62.24% | 37 | 646 |
| 9 | 6VZG_K | 813 | 62.24% | 37 | 646 |
| 10 | 6EG2_A | 621 | 91.94% | 15 | 497 |
