# Supplementary material for: Deep‐targeted gene sequencing reveals ARID1A mutation as an important driver of glioblastoma
Source: CNS Neurosci Ther. 2024 Apr 11;30(4):e14698. doi: 10.1111/cns.14698 (PMC11007544; doi:10.1111/cns.14698)
Supplement: Supplementary file 6 — Data S1 [file CNS-30-e14698-s005.pdf]

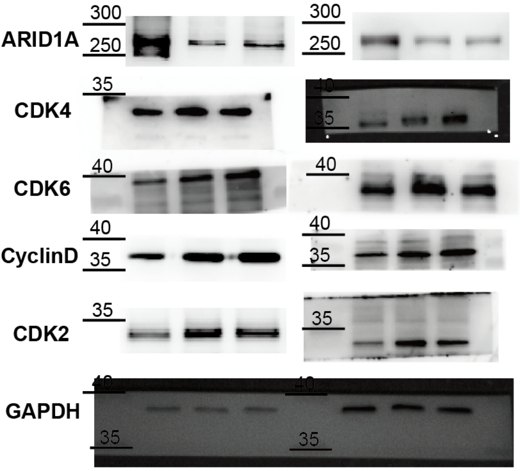

Full unedited blot for Figure 3A

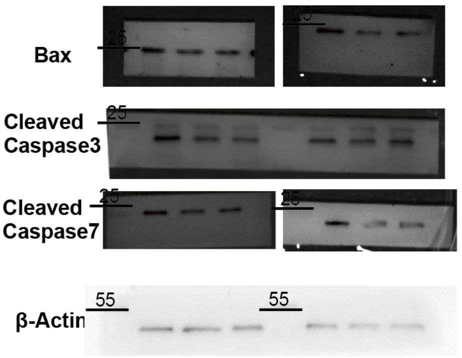

Full unedited blot for Figure 3C

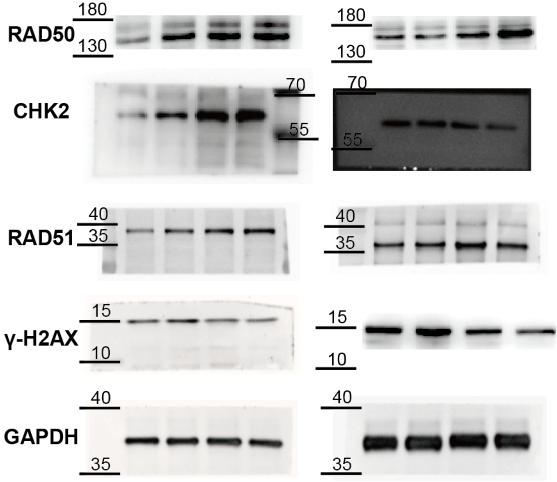

Full unedited blot for Figure 3E

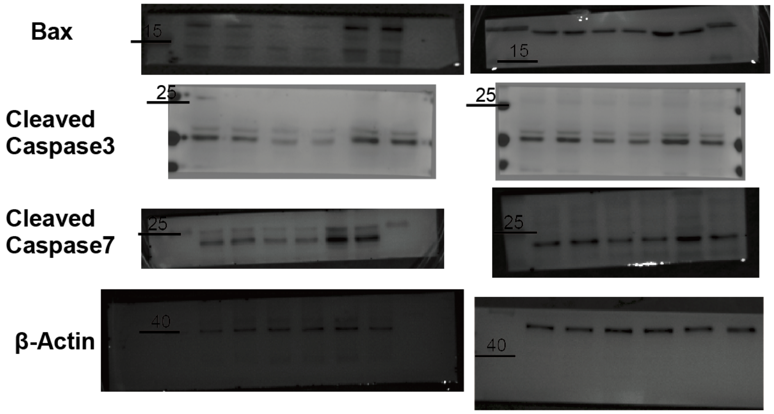

Full unedited blot for Figure 5C

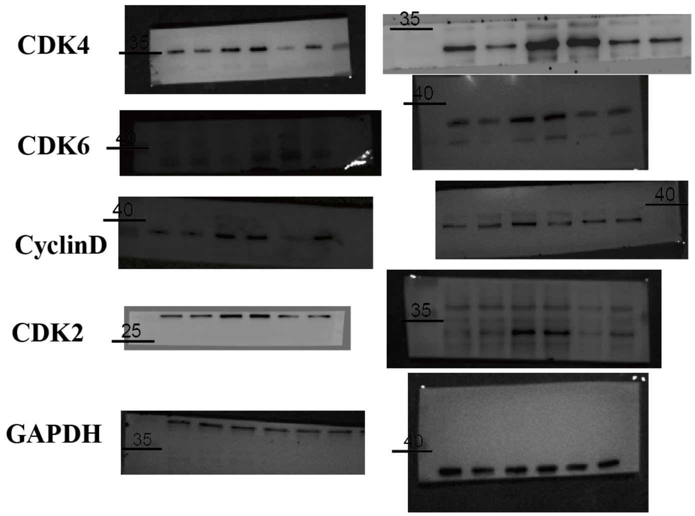

Full unedited blot for Figure 5F

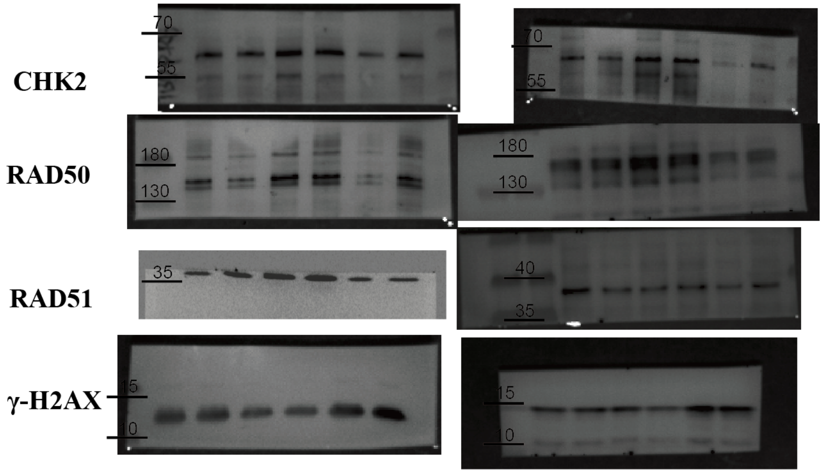

Full unedited blot for Figure 5H
